# Role of Calcium Modulation in the Pathophysiology and Treatment of Alzheimer’s Disease

**DOI:** 10.3390/ijms24109067

**Published:** 2023-05-22

**Authors:** Daniela Baracaldo-Santamaría, Sara Sofia Avendaño-Lopez, Daniel Felipe Ariza-Salamanca, Mateo Rodriguez-Giraldo, Carlos A. Calderon-Ospina, Rodrigo E. González-Reyes, Mauricio O. Nava-Mesa

**Affiliations:** 1 Pharmacology Unit, Department of Biomedical Sciences, School of Medicine and Health Sciences, Universidad del Rosario, Bogotá 111221, Colombia; daniela.baracaldo@urosario.edu.co (D.B.-S.); sara.avendano@urosario.edu.co (S.S.A.-L.); carlos.calderon@urosario.edu.co (C.A.C.-O.); 2 Medical and Health Sciences Education Research Group, School of Medicine and Health Sciences, Universidad del Rosario, Bogotá 111221, Colombia; daniel.ariza@urosario.edu.co; 3 Grupo de Investigación en Neurociencias (NeURos), Centro de Neurociencias Neurovitae-UR, Instituto de Medicina Traslacional (IMT), Escuela de Medicina y Ciencias de la Salud, Universidad del Rosario, Bogotá 111221, Colombia; mateo.rodriguezg@urosario.edu.co (M.R.-G.); rodrigo.gonzalez@urosario.edu.co (R.E.G.-R.); 4 Grupo de Investigación en Ciencias Biomédicas Aplicadas (UR Biomed), School of Medicine and Health Sciences, Universidad del Rosario, Bogotá 111221, Colombia

**Keywords:** Alzheimer’s disease, dementia, calcium channels, calcium, therapy, calcium homeostasis

## Abstract

Alzheimer’s disease (AD) is a chronic neurodegenerative disease and the most frequent cause of progressive dementia in senior adults. It is characterized by memory loss and cognitive impairment secondary to cholinergic dysfunction and N-methyl-D-aspartate (NMDA)-mediated neurotoxicity. Intracellular neurofibrillary tangles, extracellular plaques composed of amyloid-β (Aβ), and selective neurodegeneration are the anatomopathological hallmarks of this disease. The dysregulation of calcium may be present in all the stages of AD, and it is associated with other pathophysiological mechanisms, such as mitochondrial failure, oxidative stress, and chronic neuroinflammation. Although the cytosolic calcium alterations in AD are not completely elucidated, some calcium-permeable channels, transporters, pumps, and receptors have been shown to be involved at the neuronal and glial levels. In particular, the relationship between glutamatergic NMDA receptor (NMDAR) activity and amyloidosis has been widely documented. Other pathophysiological mechanisms involved in calcium dyshomeostasis include the activation of L-type voltage-dependent calcium channels, transient receptor potential channels, and ryanodine receptors, among many others. This review aims to update the calcium-dysregulation mechanisms in AD and discuss targets and molecules with therapeutic potential based on their modulation.

## 1. Introduction

Alzheimer’s disease (AD) is the leading cause of dementia in adults over the age of 65, with an estimated global prevalence of 57.4 million cases in 2019, and an expected prevalence of 152.8 million cases by 2050 [1]. It represents a major health and socioeconomic problem due to patients’ disability and loss of independence, requiring long-term care [2]. Neurofibrillary tangles, selective neurodegeneration, synaptic dysfunction, and extracellular plaques of amyloid-β (Aβ) are the hallmarks of this disease. In addition, memory loss and cognitive impairment secondary to cholinergic dysfunction at the early stages, and calcium and N-methyl-D-aspartate (NMDA)-mediated neurotoxicity at the advanced stages, are features associated with AD. Most of the attempts to treat AD have focused on these pathogenic mechanisms, including an increase in cholinergic neurotransmission with cholinesterase inhibitors (donepezil, rivastigmine and galantamine), NMDA receptor (NMDAR) antagonist (memantine) and, recently, with monoclonal antibodies directed against Aβ [3]. Taking into account that oligomeric forms of Aβ are more toxic than fibrillar forms [4], the most effective antibodies have been directed precisely against the oligomers (aducanumab) [5] and anti-Aβ protofibrils (lecanemab) [6]. According to a recent phase III clinical trial (NCT03887455) [6], lecanemab has shown better results in comparison with other molecules, and also reduced cognitive decline in patients with early AD. A possible reason for this success of lecanemab may be its administration at an earlier stage compared to other studies, as well as its good tolerability among the participants. Despite these approved treatments, no intervention has been effective in curing or halting the progression of the disease.

The calcium hypothesis in AD proposes that the dysregulation of calcium signaling occurs at early stages of the disease, and it is considered as a critical event that triggers synaptic dysfunction, mitochondrial failure, oxidative stress, and neuroinflammation, with subsequent neurodegeneration [3]. Calcium signaling has essential roles in multiple neuronal functions, such as in the proliferation, migration, and differentiation of neuronal progenitors [7]. Moreover, it is involved in neural plasticity and the release of neurotransmitters from presynaptic terminals, and acts as a second messenger in the regulation of gene expression [8,9,10]. However, altered calcium homeostasis can disrupt many functions in several cells, including astrocytes, compromising neuronal activity [11,12]. Alterations in Aβ production and/or removal are among the hallmarks of AD. The production of Aβ peptides follows a series of steps involving various enzymes within the plasma membrane. A central protein in this process is the amyloid precursor protein (APP), which is a type-I transmembrane glycoprotein with a long N-terminal extracellular region and a short C-terminal cytoplasmic tail [13]. In addition, the *APP* gene is expressed in the chromosome 21 (21q21.2), implying a relationship between AD and Down syndrome [14]. The APP suffers a series of proteolytic cleavages, which can be performed by either of the enzymes α-secretase (non-amyloidogenic pathway) or β-secretase, also referred to as the aspartyl protease β-site APP cleaving enzyme 1 (BACE1) [15]. The amyloidogenic formation pathway involves the cleavage of APP by β-secretase, which produces a membrane-bound C99 fragment and a secreted soluble APPβ (sAPPβ) fragment [16]. The C99 fragment is next cleaved by the γ-secretase enzyme, which is a transmembrane complex formed by presenilin (PS), nicastrin, anterior pharynx defective-1 (Aph-1), and presenilin enhancer-2 (Pen-2) [17]. This process ends with two different products, an APP intracellular domain (AICD), used for nuclear translocation, and the Aβ peptide [18]. The produced Aβ peptides can be of different lengths. For example, a 40-amino-acid-long peptide (Aβ_1–40_) is considered benign and physiologic, while longer peptides, mainly with 42 (Aβ_1–42_) or more amino acids, have been observed to be more aggregation-prone and pathogenic [19]. In AD, it has been reported that the ratio of Aβ production is unbalanced, favoring the appearance of longer peptides, such as Aβ_1–42_, over that of Aβ_1–40_ [20]. Although APP can be found in many cells, its main producers in the central nervous system (CNS) are considered to be astrocytes and neurons [21]. While Aβ has been thoroughly studied in pathology, its physiological role is still a matter of debate, although it has been sown to be involved in several functions such as participation in neuritic outgrowth, neuroprotection, and apoptosis reduction, the modulation of neuronal calcium release, memory enhancement, and antibacterial activity [22].

While sporadic AD (SAD), the most common form of AD, has a multifactorial component, including risk factors such as the presence of apolipoprotein E (ApoE) ε4, familial AD (FAD) due to genetic variations in *APP*, *PS1*, *PS2*, and sortilin-related receptor 1 (*SORL1*) genes [23,24]. Alterations in calcium signaling can be caused by Aβ-soluble oligomers, which are produced by the abnormal cleavage of the APP by the enzymes β-secretase 1 and γ -secretase [25]. In turn, altered calcium homeostasis can accelerate Aβ production by stimulating the proteolytic activity of β-secretase 1 and, therefore, it can potentiate neurogenerative processes early in the disease [11,12,26]. Correspondingly, PS-1 and PS-2, which are catalytic components of the γ -secretase complex, have been found to alter calcium homeostasis. For instance, neurons from *PS1_M146V_* knock-in mice (an AD animal model), demonstrate a threefold increase in inositol trisphosphate (IP3)-evoked calcium responses compared with nontransgenic controls [27].

Interestingly, over the last few years, infectious agents have been considered possible risk factors for AD, including viruses, bacteria, and parasites. The rationale behind this postulates that some pathogens may accelerate the accumulation of AD-associated Aβ plaques as part of a physiological antimicrobial response [28,29] or that the pathogen itself can cause direct damage. However, the exact mechanisms have not been fully elucidated. A recent meta-analysis based on observational studies found that the following pathogens are associated with a higher risk of AD: *Chlamydia pneumoniae*, Human herpesvirus type 6, Epstein–Barr virus, herpes simplex virus type 1 (HSV-1), and the Herpesviridae family [30]. There appears to be a relationship between the herpes virus, Aβ, and calcium dyshomeostasis. For instance, a study [31] showed that after the infection of rat cortical neurons with HSV-1, virally-induced hyperexcitability occurred, which triggered an increase in intracellular calcium levels. As a result, the processing of APP was affected, resulting in the intracellular accumulation of Aβ. This suggests that it could be possible for some infectious agents to trigger calcium dyshomeostasis and contribute to the development of AD.

A myriad of calcium channels and receptors are involved in the calcium dyshomeostasis observed in AD. For instance, NMDAR and other types of glutamate receptor [32], the voltage-gated calcium channels (VGCC) [33], and the transient receptor potential (TRP) channels, are involved in the influx of extracellular calcium, which explains the increases in intracellular calcium levels in neurons and astrocytes. In addition, the release of calcium from the endoplasmic reticulum (ER) via IP3 signaling and the ryanodine receptors (RyR), induced by amyloidosis and tauopathy, were also described. Additional intracellular pathological changes include calcium efflux from the mitochondria through permeability transition pores (PTP) [34].

The early modulation of calcium dyshomeostasis in AD could be a potential therapeutic approach to altering disease progression, or it could be used as a biomarker. Considering the development of new therapeutic agents and molecular targets that affect calcium signaling in neurons and glial cells, the aim of this review is to provide an updated bench to support understanding of the importance of calcium modulation in AD and to provide new perspectives about these mechanisms, which may be of interest to researchers working on this field.

## 2. Mechanisms for Calcium Dysregulation

In this section, we review the complex regulation of neuronal calcium signaling and the evidence supporting its dysfunction in AD. Calcium is crucial for numerous processes, including neurotransmitter release and postsynaptic activity, excitability, second-messenger cascades, gene expression, and apoptosis, among others [35,36]. Therefore, the regulation of calcium concentration in both extra- and intracellular compartments is vital for cellular homeostasis. Moreover, complex analyses pinpoint enriched calcium-mediated signaling, the serotonin-receptor pathway, and cellular responses to growth factor stimulus, among others, as key hallmarks of neuronal vulnerability factors in AD [37].

In a recent in silico study (a molecular dynamics simulation), it was shown that calcium may reduce the interaction between Aβ_1–42_ monomers in the membrane and can reduce the binding affinity to form oligomers [38]. However, other studies indicate that higher levels of calcium may facilitate Aβ-oligomer formation in comparison with fibrillar forms. In addition, it has been extensively reported that calcium may enhance the stabilization and insertion of amyloid into the lipid bilayer [39,40] and reduce the fusion between lysosome and autophagosome to degrade amyloid. This divergence is probably due to the type of amyloid studied (Aβ_1–40_ vs. Aβ_1–42_), the different biochemical effects of the calcium, depending on the site of the membrane where it interacts (intracellular vs. extracellular), the degree of affinity with specific molecules in the lipidic bilayer (i.e., cholesterol, copper, and specific amino acids), the concentrations of calcium in specific compartments (microdomains), and the time of exposure. Indeed, prolonged exposure and elevated levels of extracellular and intracellular calcium increase the phosphorylation state of tau. In contrast, when low concentrations are administered in a short-term period, a reduction in phospho-tau results instead [41]. In turn, tau itself may block the calcium efflux from the mitochondria and make neurons and astrocytes vulnerable to apoptosis and cell death at physiological intracellular calcium levels [42]. Correspondingly, in a recent study with aged monkeys, it was shown that hyperphosphorylated tau is associated with increases in calcium leakage from the ER into the cytosol, reduced calcium-binding proteins, and the impairment of cognitive performance [43]. Therefore, low levels of calcium may have neuroprotective effects, but higher levels, such as those extensively reported in AD models, can enhance the neurodegenerative effects of tau and amyloid, which, in turn, may increase calcium toxicity.

Many elements are involved in calcium regulation and signaling, such as the ER, the mitochondria, and other organelles, such as the nuclear envelope and neurotransmitter vesicles [44]. Sensors and buffers are also key to calcium signaling. These include, for instance ATP, a highly mobile and effective calcium chelator [45] and parvalbumin, a small calcium-binding protein, which has shown to be a slow calcium buffer and to mediate calcium-dependent metabolic and electric processes in GABAergic interneurons [46,47]. All these mechanisms are tightly interconnected in order to achieve a steady physiological intracellular calcium concentration, ranging from 50 to 100 nM [48].

Furthermore, it is critical to control calcium concentrations in a spatial and temporal fashion. In neurons, synaptic activity is related to receptor-operated channels, such as NMDAR, or to ionic channels, such as L-type VGCC (L-VGCC). If the intracellular calcium rises within microseconds and is localized, it might trigger neurotransmitter release [49], but if the electrical impulses are sustained over time, it might activate long-term potentiation (LTP) and gene expression [50]. Nonetheless, how exactly a specific rise in calcium can trigger a given mechanism remains to be fully elucidated. In the case of LTP, some authors propose that the spatial compartmentalization of calcium signals could link different calcium sources to the downstream effector mechanisms responsible for the maintenance of different forms of LTP [51].

As mentioned above, increased calcium ions beyond the physiological state are associated with Aβ production and accumulation, as well as with tau hyperphosphorylation [52]. Furthermore, preclinical evidence shows that Aβ can alter calcium concentrations, as it activates the IP3 receptor (IP3R) and RyR, creates membrane pores permeable to cations, and leads to cellular death [34]. Alterations in ER and mitochondrial calcium regulation are further explored in Section 3.3. As stated previously, calcium compartmentalization must be considered in order to understand the different mechanisms it can trigger. Therefore, the following sections are subdivided into presynaptic, postsynaptic, and synaptic plasticity, to better establish the pleiotropic roles of calcium-mediated signaling.

### 2.1. Presynaptic

Synaptic dysfunction arises when a functional or structural alteration disrupts synaptic connectivity, and it can be the cause or the consequence of a given pathology [53]. In AD, synaptic dysfunction arises when mutated presenilin genes, such as *PS1*, modify calcium homeostasis. Cumulative evidence shows that the proteins encoded by mutated *PS1* and *PS2* interact with IP3R, RyR, the sarco/ER calcium ATPase (SERCA), the ER, and phospholipase C, increasing the overall sensitivity to calcium or the opening probability, this inducing an increase in calcium levels in the presynaptic intracellular space [54,55]. Furthermore, postmortem human studies have shown that spine and dendritic losses are hallmarks of AD, and that they are closely related to cognitive impartment [54]. The Aβ oligomers obtained directly from the brains of AD patients were shown to impair LTP and enhance long-term depression (LTD) in mouse hippocampal slices [56]. Furthermore, the injection of these oligomers into the lateral ventricle was found to disrupt the memory of a learned behavior in normal rats [57]. Other presynaptic alterations in calcium dynamics involve the downregulation of cannabinoid receptor 1 (CB1) in the hippocampus and in the caudate-putamen [58], the activation of nicotinic acetylcholine receptors (nAchR) through the α and β subunits [59,60,61], and the hyperactivation of VGCC.

The CB1 is a Gi/o-coupled receptor that reduces cyclic AMP (cAMP) production and induces neuronal hyperpolarization through its crosstalk with potassium and calcium channels [62]. Possessing pleiotropic functions, CB1 is found in dense quantities in the presynaptic neurons of both the CNS and the peripheral nervous system (PNS), and it is especially abundant in the neocortex, amygdala, basal ganglia, cerebellum, and hippocampus [63,64]. For example, in the hippocampus, CB1 activation by 2-arachidonoylglycerol (2-AG) can modulate the presynaptic neuron to reduce neurotransmitter release by inhibiting N-type VGCC, thus attenuating postsynaptic neuronal activation [65,66]. Furthermore, it has been shown that CB1 activation is involved in neuronal survival [67]. Interestingly, postmortem human studies evidence that in patients with mild and moderate AD, there is increased efficiency of CB1, especially in the hippocampus [58,68]. This increase in CB1 efficacy might be explained as a protective and compensatory mechanism in the early stages of AD. In contrast, decreased density and efficacy of CB1 was observed in later stages of the disease [58,68]. Aso et al. [69] generated a double transgenic *APP/PS1* and CB1 knockout mice, which, after two months, died suddenly due to spontaneous seizures. In the same study, using an identical AD phenotype, but this time heterozygous for CB1, the mice showed decreased levels of postsynaptic density protein 95 (PSD-95) and accelerated memory impairment. Nonetheless, not all the evidence shows a decrease in CB1 density and function with the progression of AD [70,71]. In summary, CB1’s influence over N-type VGCC and neurotransmitter release is key to maintaining an excitatory/inhibitory balance and to preventing neural death, which suggests the possibility of evaluating cannabinoids as potential therapeutic agents in AD. 

Cholinergic neurons from the medial septum and the diagonal band of Broca areas, as well as their projections to the hippocampus, play a major role in learning and memory processes [72]. Studies on human postmortem-brain samples and neuroimages showed a massive loss of basal forebrain cholinergic neurons in AD [73,74]. The cholinergic theory of AD supports the use of cholinesterase inhibitors, which have been effective in the treatment of amnesic symptoms in their early stages [75]. In addition to the reduction in cholinergic neurons, some changes in nicotinic and muscarinic receptors have been described. The nAchR is a pentameric, cation-selective, ligand-gated inotropic receptor permeable by sodium, potassium, and calcium, and it has been related to various CNS functions and processes, such as neurodevelopment, reward, learning, and memory [76,77]. In addition, of all the α-subunit-receptor combinations, the α7nAchR subunit has been shown to strongly interact with Aβ [78,79]. Although α7nAchR subunits are located in several cell sites, many of these interactions seem to affect presynaptic function [80]. Indeed, Aβ_1–42_ blocks α7nAchR-dependent-calcium intracellular rise and, thus, acetylcholine release [79]. Furthermore, it was reported that fibrillar Aβ exerts neurotoxic effects, which are mostly mediated through α7nAchR blocking, while oligomeric Aβ allosterically modulates and activates α7nAchRs, increasing intracellular calcium concentration, thereby stimulating downstream signaling pathways and affecting synaptic function [81].

The VGCCs are further types of channel involved in the presynaptic disruption of calcium homeostasis. These voltage channels form pentameric voltage-gated receptors through the assembly of an a1 subunit with different combinations of α2, β, g, and d subunits. They can be further classified into L, P, N, Q, R, or T, depending on the current and sensitivity to toxins [82], and into the Ca_v_1, Ca_v_2, and Ca_v_3 subfamilies [83].The Ca_v_ channels, specially Ca_v_2 channels, are mostly involved in the release of neurotransmitters, hormones, and neuropeptides, in neurite outgrowth, and in the activation of calcium-dependent enzymes, such as calmodulin-dependent protein kinase II (CaMKII) and protein kinase C (PKC) [84]. In AD, compelling evidence showed that Aβ oligomers increase L-VGCC currents in a model of human neuroblastoma (MC65) cells, and that isradipine, a calcium-channel blocker, prevented the aberrant influx protecting the MC65 cells [33]. Kim and Rim [85] found that surface protein levels of Ca_v_1.3 were significantly increased by Aβ_25–35_, which also increased Ca_v_1.3 activity by interacting with the beta subunit of this channel, resulting in increased calcium entrance to the cell. Furthermore, Aβ_1–42_ was shown to increase glutamate and noradrenaline exocytosis from rats’ cortical nerve endings due to the activation of N-type calcium channels, precisely by the Ca_v_2.2 channels [86]. Therefore, several presynaptic proteins, such as CB1, cholinergic receptors, and VGCC, can be considered as potential therapeutic interventions regarding calcium regulation in AD.

### 2.2. Postsynaptic

Calcium dynamics in postsynaptic neurons are also affected in AD. Several of these pathways involve postsynaptic glutamate receptors, such as NMDAR or α-amino-3-hydroxy-5-methyl-4-isoxazolepropionic acid (AMPA) receptor, TRP canonical channels (TRPC), and L-VGCC.

Excitotoxity has been defined as neural damage secondary to the excessive release of excitatory neurotransmitters (mainly glutamate), which prompt a large amount of calcium influx into the cells [87]. This phenomenon has been described as a common pathway leading to cell death in many neuropathological conditions, such as AD, amyotrophic lateral sclerosis, traumatic brain injury, stroke, and catatonia, among others [88,89,90,91]. Memantine, an NR2B-containing NMDAR blocker, is a FDA-approved medication prescribed to patients in advanced stages of AD [92]. This drug was shown to diminish glutamate excitotoxicity and, therefore, attenuate cellular death [93].

The NMDAR-mediated calcium influx and excitotoxicity are well-described mechanisms that lead to neuronal death. In an AD triple-transgenic mice model, the over-activation of the NMDAR led to massive disruption in RyR, the ER, and calcium-induced calcium release (CICR), primarily located in the dendritic processes, spine heads, and the soma of pyramidal neurons [94]. Moreover, it was demonstrated that Aβ oligomers can affect spontaneous synaptic activity by suppressing P/Q-type calcium currents [95]. Aggregates of Aβ were shown to attach themselves to beta-1 integrin and trigger the phosphorylation and activation of NMDAR in a Src-kinase-dependent manner [96]. However, excitotoxicity is not limited to NMDAR, as calcium-permeable AMPA receptors (CP-AMPAr) can also induce it [97]. These AMPA receptors are transmembrane-cation-selective tetrameric assemblies composed of four subunits (GluA1 to GluA4). Specifically, CP-AMPAr lacks GluA2 subunits, which dictate AMPA-receptor-ion selectivity and voltage dependence [98]. Interestingly, GluA2 subunits prevent the entrance of calcium and zinc [99]. The presence of CP-AMPAr has mostly been described in early developmental stages, and its functions encompass synaptic plasticity in specific interneurons during synaptogenesis, in the formation of neuronal circuitry during early development, in heterosynaptic plasticity, and in associative learning [98,100,101]. Furthermore, an increase in CP-AMPAr levels has been proposed as a pathological marker in many neurological diseases, including AD [102]. In CA1 rats’ hippocampal slices, Aβ induced the rapid enhancement of synaptic transmission mediated by CP-AMPAr and, therefore, an increase in postsynaptic calcium and PKC [102].

In AD, neurodegenerative progression was associated with Aβ-related alterations in glutamate signaling, which may increase the appearance of excitotoxicity [103]. As previously mentioned, these toxic effects might arise from excessive calcium entry into the cell, which exceeds calcium-binding proteins’ capacity, inducing mitochondrial and ER stress [104]. The development of oxidative stress is a well-known consequence of organelle stress, which leads to neuroinflammation and cellular apoptosis, exacerbating cellular damage [105].

The TRPCs are receptor-operated calcium-permeable nonselective cation channels. Seven mammalian TRPC members contribute to a broad spectrum of cellular functions and physiological roles [106]. The TRPCs are mostly localized in the plasma membrane, but a few of them act on the membranes of intracellular organelles, such as endosomes and lysosomes [107]. The TRPCs are recognized as crucial for calcium regulation in neurons [108]. Specifically, TPRC6 is a negative regulator of NMDA-dependent calcium entry in hippocampal neurons; therefore, it is an excitotoxicity-preventing channel [109]. It is also related to the cAMP-response element-binding protein (CREB), described later in this section [110]. Interestingly, TPRC6 has been found to be both downregulated and overproduced in different AD models [111]. Exploring human embryonic kidney (HEK293) cells, Lessard et al. [112] found that PS-2 influences TRPC6-mediated calcium entry into these cells, although thy proposed the existence of an intermediary protein that leads to higher calcium influx, mediated by TRPC6. In contrast, other studies indicated that the activation of TRPC6 restored the proportion of mushroom spines in cellular models of FAD and induced LTP in hippocampal mouse-brain slices extracted from an AD model [113].

As previously stated, L-VGCCs are crucial for calcium regulation. In the cultured cortical neurons of mice, Aβ was shown to increase calcium influx through L-VGCC and neurodegeneration [114]. Interestingly, the authors of the study mentioned previously found that Aβ neurotoxicity was mediated by L-VGCC rather than by NMDAR. Furthermore, L-VGCC excitotoxicity might also arise from the activation of mitogen-activated protein kinase (MAPK) in neurons exposed to Aβ [115]. In addition, in postmortem brains from individuals with AD, the CA1 region of the hippocampus was found to be particularly susceptible to L-VGCC-mediated cellular death [116]. Therefore, early neuronal death might be associated with L-VGCC activation, particularly in the hippocampus, a key area in AD.

Increased calcium can augment oxidative stress in neurons, playing a key role in neuronal death [117]. Furthermore, an increment in the activation of NMDAR leads to a higher cytosolic calcium concentration, which, when sustained, amplifies the production of reactive oxygen species (ROS), such as peroxyl radicals (ROO⋅), the hydroxyl radical (OH⋅), the superoxide radical anion (O–2), peroxynitrite (ONOO-), single oxygen (O_2_), and hydrogen peroxide (H_2_O_2_), bypassing the antioxidant countermeasures of neurons. For example, in damaged mitochondria, ROS triggers caspases and calpains conducive to apoptosis [118]. Furthermore, oxidative stress encourages indirect tau phosphorylation through the increased formation of paired helical filaments [119,120]. Therefore, several calcium-related postsynaptic mechanisms, such as the glutamatergic NMDAR and AMPA receptors, TRPC, and L-VGCC, have been shown to be involved in pathogenic aspects of AD. Since calcium dysregulation is frequently associated with excitotoxicity, inflammation, and oxidative stress, it can be considered an important therapeutic target in neurodegenerative disorders, such as AD.

### 2.3. Synaptic Plasticity

Calcium is crucial for learning and memory codification and consolidation, as this element plays important roles in all the synaptic components (presynaptic, postsynaptic, and glial). In aged individuals, calcium dyshomeostasis has been associated with both memory and learning deficits [121]. This is also apparent in AD, where canonical findings show calcium dysregulation together with increased LTD and decreased LTP [122,123]. Furthermore, synaptic plasticity has been shown to be disrupted in AD animal models when calcium signaling is altered, compromising the function of the ER and the IP3R [124,125,126].

The CAMKII and the calcium-dependent phosphatase calcineurin (CaN) are proteins involved in LTP and LTD, respectively. The CAMKII is found in dense quantities synapses in the CA1 hippocampus, and its holoenzymes are activated when bonded to calcium [127]. In postmortem studies conducted on brains from individuals with AD, a large number of synapses was found to be lost, together with an increase in CAMKII activity, determined through immunohistochemistry [127,128]. Furthermore, tau proteins (involved in the production of neurofibrillary tangles) are known to be phosphorylated by CAMKII, which affects their structure and solubility [129,130]. On the other hand, preclinical experiments using oligomeric Aβ on human neuroblastoma SY5Y cells, transgenic *Tg2576* AD mice, and Sprague-Dawley rats, showed high activity of CaN, leading to an increase in LTD and in CaN-dependent memory deficits [131]. Moreover, another study reported that the inhibition of CaN in *Tg2576 APP* transgenic mice showed an improvement in associative learning and memory [132]. These results suggest that different calcium-dependent enzymes, such as CaMKII and CaN, may play critical roles in AD.

Regarding LTP disruption in AD, it was proven in rats that Aβ_1–42_ directly impairs LTP in CA1 and dentate gyrus regions of the hippocampus, as it inhibits NMDA activity [85,133,134]. The presence of Aβ_1–42_ alters LTP-related protein production in a dose-dependent manner, possibly through the inhibition of protein-synthesis processes or through the disruption of calcium-dependent enzymes, such as CaN [135,136]. Yeung et al. [137] reported a postmortem characterization of NMDAR in individuals with AD, using free-floating immunohistochemistry and confocal laser scanning microscopy. They found an increased expression of GluA2 in the striatum and dentate gyrus, of GluN1 in the dentate gyrus, entorhinal cortex, CA1, CA2, and CA3 hippocampal areas, and of GluN2A in CA1, in brains with AD. These results suggest the differential expression of glutamatergic receptor subunits in AD-brain regions, which may also be related to diverse region-specific calcium responses in this disease. Furthermore, Zhao et al. [138] showed, in rat hippocampal slices, how Aβ_1–42_ increased the phosphorylation of GluR1, an AMPA subunit, thus impeding LTP formation in the dentate gyrus.

Eight different subtypes of metabotropic glutamate receptors (mGluR) have been reported (mGluR1 to 8), which suggests the complex range of signals glutamate can produce on a cell. These subtypes can be further classified into three groups: group I (pre-postsynaptic and excitatory), encompassing mGluR1 and 5; group II (presynaptic and inhibitory), featuring mGluR 2–3; and group III, including mGluR 4, 6, 7, and 8 (presynaptic and inhibitory) [139]. In AD, the activation of these receptor subtypes may offer neurotoxic or neuroprotective responses. For instance, mGluR1 activation is linked to LTD and contributes to the early-stage memory and learning deficits observed in AD [140,141], while mGluR5 localization shifts towards synapses in the presence of Aβ oligomers and increases synaptotoxicity [142]. The Aβ oligomers have been shown to interact with the complex composed of mGluR5 and cellular prion protein, disrupting mGluR5 signaling and leading to synaptic failure [143]. Furthermore, while mGluR2 activation increases synaptic Aβ_1–42_ generation, its inhibition improved neurogenesis and synaptic transmission and diminished Aβ toxicity in AD-mouse models [144]. Conversely, it was shown in female adult Rhesus macaques that astrocytic mGluR3 activation protects against NMDAR-mediated excitotoxicity [145]. In addition, mGluR7 activation protects against synaptic NMDA-induced excitotoxicity, although the presence of Aβ selectively impairs this benefit [146]. As with mGluR7, mGluR8, a group III metabotropic receptor member, has been shown to dampen glutamate excitotoxicity and regulate neurotransmitter release in mouse and human neurons [147]. Furthermore, in mGluR4 knockout mice it was shown an improvement on LTP in the hippocampal CA1 region, but not in the prefrontal cortex [148].

In this section, we described alterations in neuronal excitability, plasticity, and functional networks observed in AD, emphasizing the importance of calcium in the functional regulation of synapses and cell survival. It is noteworthy that strong preclinical evidence supports the amyloidogenic hypothesis, and that it suggests that both oligomeric and fibrillar Aβ aggregates disrupt neuronal calcium dynamics. However, the mechanism through which one can be more neurotoxic than the other remains to be elucidated. Nonetheless, clinical trials with monoclonal antibodies that impede Aβ aggregation have shown only discrete efficacy, leaving the question as to the origin of pathological changes in AD open. In this regard, calcium dysregulation might be one of the critical aspects leading to AD development and, therefore, it may be considered as a salient therapeutic target. In the next sections, we examine in detail organelles’ and pumps´ functions in calcium homeostasis and its disruption in AD.

### 2.4. Calcium Transporters, Pumps, and Associated Membrane Channels

Specific calcium transporters and pumps are involved in a variety of pathophysiological processes implicated in AD, beyond neuronal excitability. Indeed, experimental evidence suggests that both the sodium–calcium exchangers (NCX) and the plasma membrane calcium-ATPases (PMCA) are involved directly and indirectly in both neurogenesis and neurodegenerative processes. For instance, the cooperation between PMCA, ionic exchangers, and calcium buffers has an impact on neuronal survival [7]. The PMCA and the NCX are considered important in calcium homeostasis, since they can transport excess calcium outside of the cell in response to an agonist that stimulates ER calcium release [149,150]. Furthermore, PMCA and NCX are negatively affected when briefly exposed to oxidative stress, resulting in rapid downregulation in the plasma membranes of hippocampal neurons [151]. Correspondingly, both PMCA and NCX appear to be compromised in nearly all neurodegenerative disorders, including Huntington’s disease, Parkinson’s disease, and AD [152], providing important targets for neural protection. These findings evidence the important roles of calcium ions in homeostasis and as second messengers in many intracellular processes, making the NCX, the calcium-activated potassium channels (KCa), and the PMCA interesting therapeutic targets for AD, worthy of exploration [153].

The NCX is an antiporter that is mainly expressed in the cerebral cortex and the hippocampus [154]. In mammals, three types of NCX have been reported: NCX1, mostly located in the heart, the brain, and the kidneys; NCX2, located in the brain; and NCX3, located in both the brain and the skeletal muscles [155]. In general, NCX appears to play a neuroprotective role in AD, especially NCX3, since its dysfunction seems to be implicated in neuronal death by caspase-12 activation, secondary to ER stress, in later stages of the disease [156]. Furthermore, these exchangers appear to have important effects in cognitive processes affected by AD, since NCX2 and NCX3 proteins and their respective mRNA levels were reduced in transgenic *APP23* and *APP-KI* mice [157]. Furthermore, in the same study, it was reported that *NCX2^+/−^* and *NCX3^+/−^* mice exhibited impaired LTP and memory-related behaviors, which were associated with CaMKII autophosphorylation processes and CaN elevated activity. Interestingly, treatment with tacrolimus (FK506), a CaN blocker, significantly restored dysfunctional hippocampal LTP in the *APP-KI* mice, suggesting a possible therapeutic intervention that warrants further studies.

The KCas are a heterogeneous group of outward-current-selective potassium channels regulated by intracellular calcium signaling. At the neuronal membrane, they are responsible for regulating the efflux of potassium, and they are therefore involved in the regulation of cellular excitability, control over intracellular ionic homeostasis, neuronal plasticity, and even the activation of microglia [158,159]. The KCa family can be functionally subdivided into three groups, based on their conductance: large-conductance channels (KCa1.1, BKCa, and BK), intermediate-conductance channels (KCa3.1, SK4, IKCa, and IK), and small-conductance channels (KCa2.1–2.3, SKCa, and SK) [160]. The large-conductance channels are activated by two important mechanisms, depending on the intracellular calcium levels and cellular depolarization, while the small-conductance channels appear to be activated only by intracellular calcium levels [161]. Regarding their specific neuronal roles, it has been evidenced that the large-conductance channels are involved in the repolarization of calcium spikes in dendrites, thus controlling the action-potential firing rate [162]. This regulation of excitability has been described in cortical L5 pyramidal neurons and hippocampal CA1 pyramidal cells [163]. The small-conductance channels are synthesized ubiquitously in the human brain and are involved in calcium regulation, alongside the NMDAR [164]. Furthermore, these channels are involved in LTP regulation in the hippocampus [165] and in the prevention of hyperexcitability in cortical neurons [166]. Yamamoto et al. [167] showed that intracellular Aβ inhibits large-conductance KCa in cortical pyramidal cells from wild-type mice. Consequently, it was reported that Aβ-induced BK suppression broadened action potentials and enhanced voltage-dependent calcium entry, thereby disrupting calcium homeostasis and inducing neuronal death [168]. Although the mechanistic explanation for how intracellular Aβ inhibits large-conductance KCa is still not clear, the possibility of improving neuronal survival through the regulation of this interaction is quite appealing.

Notably, both small- and intermediate-conductance KCas appear to be involved in sex differences regarding AD development, probably through Aβ-induced alterations in the cerebral endothelium and mitochondria, secondary to estrogen loss, in female rats [169]. Considering the functional and structural alterations evidenced in the neurovascular coupling induced by calcium dysregulation in AD, it was shown that SKA-31 (an activator of both SKCa and IKCa) appears to have beneficial cardiovascular effects by improving vasodilation and restoring *KCNN3*- and *KCNN4*-gene expression without immune activation in rats [170]. Further studies are needed to determine whether the beneficial effects of SKA-31 could be extended to AD by improving vasodilation and reducing the neurovascular unit (NVU) and blood–brain barrier (BBB) alterations observed in the disease. In addition, these channels appear to be involved in microglial activation, since it was observed that the knockdown of its expression reduced microglial response to lipopolysaccharide administration via the toll-like receptor 4 (TLR4) and the nuclear factor kappa-light-chain enhancer of activated B cells (NF-κB) pathway in murine models [171]. Interestingly, microglial KCa3.1s are undergoing studies as potential therapeutic targets for AD, since their blockade appears to have neuroprotective and anti-inflammatory effects [172].

The PMCAs are part of the P2B branch of the P-type ATPase superfamily, with a wide variety of types expressed in the brain, which regulate calcium extrusion from cells [173]. Both PMCA2 and PMCA3 are considered to be neuron-specific and play an important role in calcium homeostasis, since they are the most sensitive cellular calcium detectors [174]. Furthermore, they have important physiological functions in the CNS, such as the regulation of neural development and sensory processing [175]. The PMCA decrease function with aging, and this has been associated with age-dependent intracellular calcium alterations, which include both increased levels of intracellular calcium and longer periods of calcium elevation after neuronal stimulation, which can augment neuronal vulnerability to stressors [176]. The PMCAs have an important role in mediating extracellular calcium exit from cells, in conjunction with NCX, due to their high calcium affinity and low transport capacity, in contrast to the high capacity and low affinity that NCX displays [177]. In AD, PMCAs appear to be inhibited by the presence of Aβ deposits, although the mechanism behind this is not clear. Interestingly, the same authors reported a protective effect of cholesterol on Aβ-mediated PMCA4 inhibition [178,179].

The exchangers and pumps presented in this section can have important effects on the modulation of intracellular calcium levels, with both neuroprotective and neurotoxic effects. The expression of some NCX isoforms appears to have important and long-lasting effects on hippocampal LTP and cognitive deficits, which involve CaMKII autophosphorylation and CaN activity. Therefore, the amelioration of these alterations via CaN blockers, such as FK506, has therapeutic potential. Further studies could explore the possibility of using other CaN blockers in order to evaluate the effects on memory deficits and other cognitive alterations secondary to hippocampal dysfunction in AD. In addition, BKCas have shown an important role in modulating the repolarization of calcium spikes in dendrites in both cortical and hippocampal neurons. This suggests the possibility of reducing excessive local calcium levels by targeting these exchangers, with a possible reduction in neurotoxic effects, such as hyperexcitability, neuroinflammation, and Aβ accumulation. On the other hand, regarding the possibility of improving neurovascular dysfunction, activators of SKCa and IKCa, such as SKA-31, have shown interesting cardiovascular benefits with little immune-system activation, suggesting that they could alleviate some of the microvascular alterations that compromise the NVU and BBB described in AD. However, this therapeutic possibility needs to be experimentally tested in specific models of the disease. Finally, considering the importance of the PMCA in the regulation if calcium homeostasis, an interesting association was found between cholesterol levels and PMCA function. Since the ApoE is involved in the metabolism of brain cholesterol, and alleles of the *APOE* gene encoding variant ApoE-ε4 protein are present in more than half of AD patients, and have long been considered strong risk factors for developing the disease, it could be useful to explore whether PMCAs are differentially affected by *APOE* alleles.

### 2.5. Astrocyte and Calcium Dynamics

Astrocytes play a major role in brain function and cognitive performance, controlling synaptic activity, neurovascular coupling, and metabolic supply to neurons. In addition, astrocytes regulate the CNS extracellular microenvironment by adjusting ionic levels of potassium and sodium, pH, and osmolality. Many astrocyte functions depend on intracellular calcium elevations, both in single cells and across glial networks [180].These calcium changes occur in a spatially and temporally specific manner [181] and involve global and local elevations in the perisynaptic astrocytic processes (also known as calcium microdomains) [182]. Intracellular calcium increases may be spontaneous or responses to neuronal and synaptic activity through glutamate and cholinergic systems (involving NMDAR, mGluR2, and mAChR) [181,183].

Several sources may explain calcium transients in astrocytes, which may be grouped into two categories: (1) extracellular, through either plasma-membrane calcium channels (i.e., TRPCs) or specific neurotransmitter receptors; and (2) intracellular stores from mitochondria [184,185] and ER [186]. The accumulation of Aβ and tau proteins may induce several functional impairments in astrocytes, such as reduced phagocytic activity in peri-plaque dystrophic synapses and mitochondrial dysfunction [187,188]. Most of those alterations appear due to inadequate calcium homeostasis. Indeed, Aβ disrupts gliotransmission, modifying astrocyte calcium transients through α7nAChR [11], and generates astrocytic hyperactivity with calcium waves through purinergic P2Y1 receptors [189]. The use of P2Y1 antagonists, such as MRS2179, was shown to normalize astrocytic and neuronal network dysfunction and improve cognition in AD transgenic mice [190].

A correlation between the astrocyte-calcium dysregulation induced by Aβ and inflammatory activity has been described. For instance, pro-inflammatory activity in astrocytes was associated with the signaling of the calcium-CaN/nuclear factor of the activated T-cell (NFAT) pathway in aged-related neurodegenerative conditions [191]. A study on postmortem human brain tissues showed that the activation of this pathway is associated with cognitive impairment in subjects with mild cognitive impairment (a prodromal stage of AD), as well as in subjects with severe dementia and AD. Moreover, the activity of the NFAT pathway was associated with amyloid-induced excitotoxicity in primary rat-astrocyte cultures [192]. The intracellular calcium levels in astrocytes might be affected by amyloid-induced oxidative stress (due to the production of reactive oxygen and nitrogen species), which in turn, is associated with inflammation via NF-κB signaling and glutamate excitotoxicity through NMDAR [105]. A recent study with astrocytes derived from human-induced pluripotent stem cells (iPSCs) obtained from AD patients, reported an increase in calcium transients in response to glutamate, hypertrophy, and elevated glial fibrillary acidic protein (*GFAP*) expression due to monomeric and aggregated tau. In comparison with SAD samples and controls, FAD astrocytic secretomes have a lower ability to degrade tau. Moreover, astrocyte-conditioned media led to changes in the calcium dynamics of cells, depending on whether the conditioned media were derived from SAD or FAD samples, as the astrocytes from the SAD displayed more robust intracellular calcium oscillations than those from the FAD, and neurons cultured with a conditioned medium from SAD astrocytes showed higher levels of calcium transients in response to glutamate than those cultured with astrocytes from the FAD [193].

As mentioned previously, astrocytes play a central role in CNS-homeostasis regulation, which is deeply related to functional calcium pathways. In AD, calcium dysregulation and signaling deficits have been observed in astrocytes [194]. Therefore, interventions involving calcium pathways and metabolism in astrocytes can be considered as novel and viable therapeutic targets for AD. For instance, astrocytes express the *CASR* gene encoding the calcium-sensing receptor (CaSR) [195], a cell-surface family C metabotropic receptor, which signals via G-proteins and β-arrestins and is important for the sensing of extracellular calcium levels [196]. The activation of CaSR stimulates MAPK function and calcium release from intracellular stores [197]. This receptor possesses an extracellular domain, termed *Venus flytrap*, which binds Aβ, as well as other agonists, such as amino acids (tryptophan and phenylalanine), polyamines, and aminoglycoside antibiotics [198]. Furthermore, inflammatory cytokines, such as IL-1β and IL-6, upregulate *CASR* expression [199]. Increased *CASR* expression was observed in AD animal models and in cultured neural cells [200,201]. Considering that both abnormal Aβ production and neuroinflammation are hallmarks of AD, the allosteric regulation of CaSR has been tested as a novel therapeutic target in preclinical models of AD. A CaSR allosteric antagonist (calcilytic), NPS 2143,was shown to attenuate Aβ_1–42_ secretion in both human cortical astrocytes and HCN-1A neurons [202]. Furthermore, in the same study, it was reported that treatment with NPS 2143 reduced the total CaSR protein complement, transiently raised proteasomal chymotrypsin activity, and blocked excess nitric oxide (NO) production in human astrocytes. The reduction in Aβ_1–42_ induced by NPS 2143 in human astrocytes can be explained by a shift in the α-secretase cleaving of APP, favoring the non-amyloidogenic pathway over the amyloidogenic pathway [203]. Furthermore, the treatment with NPS 2143 reduced the secretion of IL-6, intercellular adhesion molecule-1 (ICAM-1), regulated upon activation, normal T-cell expression and, presumably, secretion (RANTES), and monocyte chemotactic protein (MCP)-2 in Aβ_25–35_-treated human astrocytes [204]; other cytokines, such as IL-1β, IL-3, IL-8, and IL-16, were not affected by the NPS 2143, suggesting an Aβ-CaSR-independent pathway for these proteins.

The TRPC6 is located in many organs, brain regions, and CNS cells, including astrocytes [205]. It can be regulated by calcium/calmodulin pathways, as well as by protein serine and tyrosine phosphorylation [206]. Several important functions, such as neuronal survival, synaptogenesis, learning, and memory have been associated with TRPC6 [207,208]. It seems to play a role in AD, as mRNA blood levels of *TRPC6* were found to be reduced in patients with AD and in individuals with mild cognitive impairment [209]. Furthermore, a positive correlation between *TRPC6* mRNA blood levels and mini-mental-state-examination scores, and an inverse correlation between *TRPC6* mRNA blood levels, clinical dementia ratings scores, and index for activities of daily living (ADL) scores, were reported in AD patients [210]. Astrocytes express *TRPC6*, and it has been reported that the treatment of these glial cells with the proinflammatory cytokine IL-1β in mice induced an increase in the levels of TRPC6 and in the receptor-operated calcium entry (ROCE) mechanism, leading to the dysregulation of calcium homeostasis [211]. Hyperforin, a TRPC6 agonist obtained from the *Hypericum perforatum* plant, was shown to reduce Aβ levels and improve behavioral performance in AD animal models [106].In addition, hyperforin elevates intracellular calcium concentrations by activating TRPC6 without activating other isoforms, such as TRPC1, TRPC3, and TRPC4 [212]. Hyperforin has been shown to affect the functional profiles of astrocytes, as mice with an induced ischemic stroke exhibited an increase in astrocytic IL-6 when treated with hyperforin, which was crucial for increasing neural precursor-cell proliferation, neuronal differentiation, angiogenesis, and functional recovery after stroke [213].

The targeting of CB may represent another novel approach for AD treatment involving calcium pathways in astrocytes. The two types of CB, CB1 and CB2, can be localized in astrocytes [214]. The CB1 type can be found under physiological conditions both in the plasma membrane and in the mitochondria [215,216], and the activation of CB1 in astrocytes leads to an increase in intracellular calcium levels from internal stores [217]. In addition, the endocannabinoids released by neurons elevate calcium and stimulate glutamate release in astrocytes [218]. However, the presence of CB2 under physiological conditions in astrocytes remains controversial, although it was been shown under pathological conditions, such as amyloid triggered neuroinflammation [219]. Furthermore, astrocytes have been shown to be directly involved in the metabolism of the endocannabinoids anandamide and 2-AG [220,221]. Several findings in animal models and in humans have shown alterations in endocannabinoids, along with the presence of both CB1 and CB2 in AD, involving glial cells (for review see [214]).

In general, Aβ and tau affect the intracellular calcium levels in astrocytes, compromising several functions, such as the physiological roles of astrocytes at the synaptic level, neurovascular coupling, and the interaction with neurons, microglia, and oligodendrocytes. However, it remains to be elucidated whether calcium changes in astrocytes are causes or consequences of AD pathology. Nonetheless, several novel agents, such as CaSR antagonists, TRPC6 agonists, and cannabinoids, may be considered as promising therapeutic agents targeting astrocytes in AD (see below).

### 2.6. Mitochondrial and Calcium Dynamics

Advances in genetics and a growing understanding of molecular mechanisms have helped to unveil the association between impaired mitochondrial function, calcium dysregulation, and multiple neurodegenerative disorders, including AD. Given the important role of mitochondrial function in maintaining ionic gradients and generating synaptic membrane potentials, it is not surprising that the brains of AD patients show impaired mitochondrial function, resulting in reduced ATP levels and increased ROS production [222,223]. However, it has not yet been established whether mitochondrial dysfunction leads to AD, or whether it is a consequence of Aβ and pathological tau accumulation [223].

Mitochondrial function greatly depends on intracellular and mitochondrial calcium levels, since calcium directly stimulates enzymes in the tricarboxylic acid (TCA) cycle and the electron-transport chain (ETC), affecting cellular ATP production [224]. The overload of mitochondrial calcium concentration impairs key mitochondrial functions, leading to mitochondrial dysregulation and, thus, increases in oxidative phosphorylation and the overproduction of ROS, processes that take place in AD [223,224,225,226]. In fact, a study on *C. elegans* demonstrated that mitochondrial calcium-induced ROS production suppresses normal mitochondrial functions and promotes neuronal dysfunction [227]. Hence, adequate calcium regulation is essential for normal cellular function and depends on orchestrated machinery and inter-organelle contact, since multiple systems operate to counterbalance cytosol-calcium increase [228].

The mitochondrial calcium uniporter (MCU) complex is the principal pathway allowing calcium influx into the mitochondrial matrix. This inner mitochondrial membrane (IMM) uniporter is highly selective for calcium ions [223,229]. It responds to the augmentation of extramitochondrial calcium levels and, therefore, represents a crucial structure for mitochondrial calcium-buffer function [223]. Electron transfer during ATP production creates an electrochemical gradient across the IMM; the mitochondrial membrane potential (∆Ψm) represents this potential difference and provides a driving force to uptake cytosolic calcium into the mitochondrial matrix, via the MCU complex [230]. The response of the MCU depends on regulatory subunits, called mitochondrial calcium-uptake proteins (MICU), located in the intermembranal space. The MICUs act as gatekeepers, as they prevent calcium overload at low extramitochondrial concentrations [223,231]. In particular, MICU3 acts as an enhancer of mitochondrial calcium uptake in the nervous system [231,232]. Any changes to this uniporter or its subunits result in the disruption of calcium levels [233]. In fact, findings in mouse AD models demonstrated that blocking the MCU complex reduces mitochondrial calcium levels [227,234]. For instance, a study using a mouse model of AD showed that increased mitochondrial calcium levels preceded neuronal dysfunction, and suggested that blocking calcium uptake by inhibiting the MCU complex might represent a novel therapeutic strategy [234].

The voltage-dependent anion-selective channel (VDAC), or mitochondrial porin, is the most abundant protein in the outer mitochondrial membrane (OMM), which allows the entry of small molecules (<10 kDa) and ions, including calcium, into the mitochondria [235,236]. In addition to its role as a metabolite exchanger between the cytoplasm and the mitochondria, the VDAC can engage in protein–protein interactions, most notably with the IP3R [236]. 

In excitable cells, the primary mechanism in the extrusion of calcium from the mitochondria into the intracellular space is the mitochondrial NCX (mNCLX) [237]. However, during strong depolarization, and in the presence of high intracellular sodium concentrations, mNCLX can reverse its mode of operation by mediating calcium’s influx into the mitochondrial matrix [228]. A decreased level of this exchanger was reported in AD human brains [238]. Furthermore, animal models of AD (*3xTg* AD-mouse model) showed neuronal damage associated with mitochondrial calcium overload due to impaired calcium efflux [238]. Other studies suggest tau-induced alterations in calcium efflux through mNCLX, as well as an increased vulnerability to calcium-induced cell death [42]. The mNCLX also plays an important role in cellular energy production, since calcium levels appear to have both neuroprotective and detrimental effects on the mitochondria [239]. Moreover, the ubiquitous hydrogen/calcium exchanger (mHCX) works as a second mechanism for the extrusion of excessive mitochondrial calcium levels, and it is also known as the leucine zipper [223,226]. Thus, both mNCX and mHCX, which are involved in the extrusion of elevated calcium levels in the mitochondrial matrix, might be proposed as therapeutic targets that could modulate calcium dysfunction in AD.

Defects in mitochondrial dynamics might also be implicated in the pathogenesis of neurodegeneration [240]. These disturbances involve subcellular trafficking, inter-organellar communication, mitochondrial fission and fusion, and mitochondrial quality control [227]. Fission and fusion regulate cytosolic calcium concentrations, which have been linked to neurotransmitter release and terminal axon branching [241]. In particular, ApoE-ε4 has been associated with the downregulation of mitochondrial dynamics, decreasing fusion and fission proteins, especially in older individuals [242]. Additionally, neurons that presented ApoE-ε4 had increased ROS and mitochondrial calcium levels, and impaired the ATP-generation capacity, suggesting a pathological effect of this protein [243]. A study on young adults (20–39 years old) showed that non-demented ApoE-ε4 carriers have glucose hypometabolism in regions affected by AD, which may be related to mitochondrial dysfunction [244].

The trafficking of mitochondria is mediated by Miro proteins, and it is partially regulated by calcium. A study conducted on rat H9c2 cells showed that increases in calcium can disturb mitochondrial trafficking along the microtubules [245]. Moreover, a study on mouse hippocampal neurons demonstrated that the elevation of calcium influx into the mitochondria was linked to mitochondrial trafficking speed [246]. In addition, mitochondrial morphology, distribution, and function are altered in the cortex and hippocampus of patients with AD, as decreased mitochondrial networks, together with fragmented and small mitochondria, are associated with neurodegeneration [240,247]. In addition, further evidence suggests an association between the hyperphosphorylation of tau protein and the impaired trafficking of mitochondria, which endangers the viability of cells [228].

Mitochondrial quality control is crucial for adequate mitochondrial and cellular function. Thus, autophagy, a cellular catabolic process that regulates the turnover of proteins, organelles, and cellular waste, is a vital pathway for maintaining homeostasis. Due to their functional and metabolic characteristics, this process is especially important in neurons [224,230]. In a FDA-PS mouse model, researchers demonstrated the major role of calcium signaling in autophagy, which is linked to PS-2 and its ability to deplete ER calcium content [230]. Several studies have shown the accumulation of damaged mitochondria in AD, suggesting an alteration in mitophagy (the elimination of “worn-out” mitochondria via autophagy), although the exact pathogenic mechanisms are unknown [240,247,248]. Tau- and Aβ-induced dysfunction is linked to impairment in cytosolic calcium regulation, which compromises mitochondrial calcium-buffering capacity and leads to neuronal death [226,230,249]. These findings reinforce the causative role of disturbed mitochondrial quality control in AD pathogenesis.

Under physiological conditions, the mitochondrial PTP (MPTP) allows the transient diffusion of calcium and ROS [250]. This large-conductance channel traverses the IMM and the OMM and allows the exit of metabolites with molecular weights of up to 1.5 kDa [251,252]. However, in the presence of sustained elevated mitochondrial calcium levels, the prolongated opening of MPTP leads to a loss of ΔΨm, mitochondrial matrix swelling, and membrane-cristae remodeling, resulting in calcium discharge and the efflux of cytochrome c and pro-apoptotic agents, such as the apoptosis-inducing factor (AIF), from the intermembrane space [253,254]. The MPTP formation is calcium-dependent, and it is regulated by several processes, including the translocator protein (TSPO), the interaction of the outer membrane VDAC, the inner membrane channel ATP-ADP translocase (ANT), and cyclophilin D (CypD); the latter is considered to be the primary mediator of MPTP activation, and it is able to interact with Aβ [255,256]. A reduction in CypD levels and, therefore, the inhibition of MPTP, led to the prevention of cognitive and synaptic impairment in AD-mouse models [250,256]. In AD, Aβ aggregation increases mitochondrial membrane permeability and cytochrome-c release, triggering MPTP opening, which leads to apoptosis (programmed cell death) [257,258]. Moreover, it was shown that fibroblasts obtained from patients with SAD present the persistent activation of MPTP and mitochondrial calcium dysregulation [259]. Hence, targeting MPTP might represent a promising therapeutic strategy in AD, especially through the inhibition of CypD.

Several studies have shown mitochondrial dysfunction in in vitro models of AD, with consequent metabolic, oxidative-stress, and pro apoptotic mechanisms, which affect many cells in the CNS, including astrocytes [257]. These alterations involve the overall mitochondrial calcium dynamics, particularly through transmembrane calcium gradients. Indeed, Aβ may generate the opening of MPTP, with a subsequent increase in calcium uptake [257,258]. The effects of amyloid, tau, and oxidative stress on mitochondria may also affect other organelles. In fact, there is a functional complex, called mitochondria-associated membranes (MAMs), in which ER and mitochondria interact in a combined membrane network (see Section 3.4 for a detailed description). This interaction involves calcium dynamics via IP3 and RyR, located in the ER, and VDAC in the ER–mitochondria connections [260]. Recent evidence confirmed several pathological mechanisms of MAM dysfunction in AD, which include, among others, calcium signaling [261]. In astrocytes, MAMs are associated with proinflammatory signaling pathways and autophagy in the pathological context of inflammatory brain diseases, such as AD [262]. Therefore, the regulation of MAMs can also be considered as a potential target to reduce neurodegeneration [263].

In summary, adequate mitochondrial function is highly dependent on appropriate calcium regulation; thus, mitochondria have specialized machinery to maintain calcium homeostasis. The disruption of any of the relevant transporters or membrane exchangers is evidenced in animal models of AD. In particular, MCU has been extensively studied in recent years, and its modulation might represent a promising therapeutic strategy in AD and other neurodegenerative diseases. In addition, mitochondrial dysfunction and mitochondrial calcium dysregulation have been observed in brains from individuals with AD, but whether these alterations occur concurrently with amyloid-plaque formation is unclear. Regarding the altered mitochondrial dynamics in neurodegeneration, advanced combined microscopy/functional studies might be crucial for obtaining knowledge about the role of calcium regulation.

### 2.7. Endoplasmic Reticulum and Calcium Dynamics

The ER is the main intracellular calcium-storage organelle, with a concentration that is about a thousand times higher than cytosolic calcium levels [264,265]. To counterbalance excess cytosolic calcium concentrations, calcium is taken up by the ER via SERCA, at the expense of ATP hydrolysis [228]. The SERCA-mediated dysregulation of calcium has been linked with multiple neurological disorders, including AD [266]. In fact, studies suggest that the modulation of SERCA levels regulates Aβ levels [267]. Other studies revealed that SERCA blockers induce calcium dysregulation and the activation of pro-apoptotic components, leading to apoptotic cell death; a similar finding was observed after the incubation of cortical cultures with A𝛽_1–40_ or A𝛽_25–35_ peptides [268]. In addition, SERCA has been studied in the context of FAD, in which aberrant autophagy and altered calcium handling have been linked with abnormal functions of PS-1 and PS-2 [267,269]. Nonetheless, recent studies highlighted certain discrepancies in the roles of PS-1, PS-2, and ER-calcium handling. For example, mutant *PS2* in FAD (FAD-*PS2*), but not FAD-*PS1*, showed inhibitory action on the SERCA pump, leading to partial ER-calcium depletion [269,270]. Additionally, mutated genes encoding for PS are associated with upregulated levels of IP3R, which might imply abnormal calcium homeostasis [27,265].

The expression of FAD-*PS* has also been linked to alterations in store-operated calcium entry (SOCE), an essential pathway for replenishing calcium stores [270,271]. The activity of SOCE is mediated by a calcium sensor located in the ER membrane, called the stromal interaction molecule (STIM1/2), and the plasma-membrane pore-forming subunit ORAI (ORAI 1/2/3) [272]. The depletion of ER calcium leads to STIM oligomerization and the subsequent recruitment of ORAI subunits, creating calcium-release-activated calcium (CRAC) channels [273]. Studies have shown that the expression of FAD-*PS1* and *PS2* decreases the levels of SMTI1, resulting in the reduction of SOCE-mediated calcium influx [270]. This inhibition leads to instability in dendritic spines and enhances amyloidogenesis [272]. The STIM2 is predominantly located in neurons, where its principal role is suggested to be the regulation of resting levels of ER calcium and calcium leakage [274]. Additionally, it forms complexes with ORAI2 and TRPC6 to regulate SOCE in mouse models. It was suggested that it plays an important function in the maintenance of dendritic spines and memory storage [275]. In addition, growing evidence suggests that ORAI1 and STIM2 may be implicated in the pathogenesis of neurodegeneration [276]. On the other hand, a recent study showed that the downregulation of ORAI2 increases SOCE activity and decreases Aβ_42_ accumulation [277].

In the ER, NCX3 plays a major role as an ionic regulator by delaying ER stress and apoptotic neuronal death, since it mediates sodium-dependent calcium influx and increases ER-calcium refilling [154,156]. Studies in which *NCX3* was knocked out showed decreased ER-calcium concentrations, caspase-12 activation, and neuronal death [154].

The ER modulates calcium levels through IP3R and RyR. The signaling of IP3 is necessary for mitochondrial calcium supply from the ER, a process regulated by the σ1 receptor (σ1R), an ER protein implicated in neuroprotection and neuroplasticity [228,248]. The IP3R activates ER-calcium release, and it is regulated by calcium in a biphasic manner: stimulatory at low calcium levels, and inhibitory at high calcium levels [257]. On the other hand, RyR activity can be mediated by calcium itself, and it can amplify the function of IP3R through a CICR mechanism, which may deplete ER-calcium levels. All three RyR types (RyR1, RyR2, and RyR3) are present in the brain [278], although their tissue distributions are different. The RyR2 is mostly localized in the cerebral cortex, the cerebellum, and in the dentate gyrus of the hippocampus, whereas the RyR3 is found mostly in the hippocampal CA1 pyramidal cell layer, the basal ganglia, and olfactory bulbs [278]. Several animal and human models have shown the increased activity of RyR2 in AD and mild-cognitive-impairment brains [279] and a fivefold increase in RyR2 in the brains of *3xTg*-AD mice compared to controls [280]. Another study found an increase in the transcription of the *RYR3* in the cerebral cortex and hippocampus of *TgCRND8* mice brains [281].

Cultured neurons from *3xTg*-AD-mouse-knock-in mutant *PS* producing mutant *APP* revealed increased RyR-mediated calcium release [282]. Additionally, AD-mouse models revealed altered RyR function and expression, as well as increased intracellular calcium concentrations, due to CICR via the RyR [283,284]. Moreover, the use of the RyR blocker, dantrolene, was shown to maintain intracellular calcium homeostasis [283,284]. Researchers demonstrated that dantrolene binds to amino acids 590–609 of RyR1 [285], as well as the amino acids 601–620 of RyR2, particularly to the FK506-binding protein (FKBP or calstabin) subunits. These findings suggest that RyR overactivation plays an important role in ER-calcium signaling in AD [269].

Communication between mitochondria and ER is crucial for calcium homeostasis. Hotspots, or calcium microdomains, are transient regions with high calcium concentrations (>10 μM) and can be formed in the spaces in which mitochondria are juxtaposed to ER calcium channels [254]. In neurons, they are essential for controlling neurotransmitter release [223,254]. These two organelles physically interact to form functional complexes, called MAMs [225,228,240]. These MAMs, located in the perinuclear regions of cells, are crucial for maintaining multiple metabolic processes, protein transport, mitochondrial fusion and fission, apoptotic signaling and, thus, cellular integrity [229,286]. Growing evidence shows a possible association between MAM alteration and AD pathophysiology [286]. The APP accumulates in these membranes, encouraging the formation of multiple ER–mitochondria contact sites, resulting in mitochondrial calcium overload [230,287]. Various proteins have been associated with MAMs, such as PS-1 and PS-2, RyR, and IP3Rs [228,240]. Presenilins might have a role in maintaining normal ER-calcium levels, acting as low-conductance ER-leakage channels [228]. Studies show that *FAD-PS* mutations might lead to the malfunction of these ER calcium-leakage channels [223]; nevertheless, it is not clear whether mutant *PS* reduces or increases ER calcium levels [270,288,289]. Additionally, the dysregulation of calcium mediated by *PS* mutation and characteristic AD histopathological lesions has been linked to alterations in the activity of RyRs [269].

In MAMs, a functional complex is formed between VDAC and IP3R through the glucose-regulated protein 75 (Grp75) chaperone, to regulate calcium transfer from the ER to the mitochondria, suggesting its participation in calcium dysregulation in AD pathophysiology [286,290]. Recent studies indicate that upregulated MAMs might be pathogenic indicators in AD, since this feature is present in both FAD and SAD [228]. Increases in VDAC1 expression have been linked to the cortexes of postmortem AD brains in aged amyloid APP mice, and neuroblastoma cells exposed to Aβ oligomers [291]. Another study revealed an increase in not only VDAC1, but also IP3R expression in response to nanomolar concentrations of Aβ. This upregulation led to an increase in ER–mitochondria contact sites and mitochondrial calcium concentrations [292]. Additionally, several studies evaluated the association of IP3 and the presence of aberrant proteins in AD. For example, Demuro et al. demonstrated that Aβ oligomers increased IP3 and elevated cytotoxicity [293]. In contrast, *3xTg*AD mouse models showed that reduced IP3R levels attenuated Aβ accumulation and tau hyperphosphorylation, ameliorating memory deficits [294]. Moreover, several findings suggest that ApoE-ε4 may upregulate MAM activity by measuring MAM-mediated phospholipid and cholesteryl esters synthesis, supporting the role of enhanced MAM function in the physiopathology of AD. The presence of ApoE-ε4 compared to those carrying ApoE-ε3 presents an increased risk of developing AD. A study using an astrocyte-conditioned media (ACM) model showed that the MAM function was significantly increased in the cells treated with ApoE-ε4 ACM compared to those treated with ApoE-ε3 [295].

Through the complex interplay between its membrane components and its interaction with the mitochondria, the ER has multiple mechanisms for maintaining adequate calcium cytosolic levels. Alterations in the calcium-buffer capacity of ER are detected in AD. Presenilin mutations in FAD contribute to the dysfunction of these mechanisms, which includes altered calcium uptake by SERCA and SOCE, the latter representing a promising therapeutic target in AD. On the other hand, the upregulation of MAMs is another feature seen in AD. Furthermore, many of its components, such as IP3R, RyR, and VDAC1, are crucial for calcium handling in neurons, making them further interesting targets in AD.

**Figure 1:**
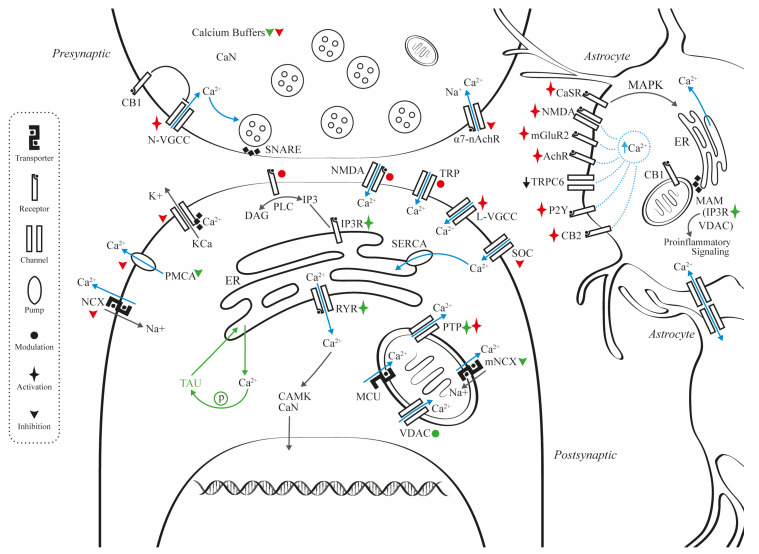
Schematic representation of cellular systems that affect calcium levels at presynaptic and postsynaptic levels, as well as in astrocytes. Main surface-membrane proteins, such as calcium transporters and receptors, as well as their interactions with several organelles and buffers, reveal a complex and coordinated system that balances the intracellular concentration of calcium. The effects of amyloidosis and tauopathy on calcium dysregulation during AD progression are illustrated. Red symbols represent the effects of amyloid on several membrane proteins; green symbols illustrate the effects of tau protein and their interaction with calcium systems at intracellular level, and blue arrows represent the direction of calcium flow in different compartments. Letter P inside the circle represents phosphorylation. Black arrows represent activation, and bars represent inhibition. The symbol ↓ in TRPC6, represents reduction in their expression. Abbreviations: calcium-dependent phosphatase calcineurin (CaN), calcium-sensing receptor (CaSR), cannabinoid receptor (CB), diacylglycerol (DAG), inositol trisphosphate (IP3), inositol trisphosphate receptor (IP3R), L-type VGCC (L-VGCC), metabotropic glutamate receptors (mGluR), mitochondria-associated membranes (MAMs), mitochondrial NCX (mNCLX), mitochondrial calcium uniporter (MCU), N-methyl-D-aspartate receptor (NMDA), nicotinic acetylcholine receptors (nAChR), permeability transition pores (PTP), phospholipase C (PLC), plasma-membrane calcium-ATPases (PMCA), purinergic receptors (P2Y), ryanodine receptors (RyR), sarco/ER calcium ATPase (SER-CA), soluble N-ethylmaleimide-sensitive adhesion receptors (SNARE), sodium–calcium exchanger (NCX), store-operated Ca2+ entry (SOCE), transient receptor potential (TRP), TRP canonical channels (TRPC), voltage-gated calcium channels (VGCC), voltage dependent anion selective channel (VDAC).

A summary of the main calcium pathways affected by amyloid and tau at the presynaptic and postsynaptic levels, as well as in astrocytes, is illustrated in Figure 1.

## 3. Novel Studies Involving Calcium Channel Modulation

### 3.1. Potential Therapies Targeting NMDAR

Because of the important role of NMDAR in the pathophysiology of AD, multiple antagonists have been evaluated for its treatment. Memantine is currently indicated for the treatment of moderate-to-severe AD. However, limited evidence of its clinical benefits has been shown [296]. Novel NMDAR antagonists have emerged with similar but distinct pharmacological properties. For example, RL-208, a novel voltage-dependent, moderate-affinity, non-competitive NMDAR blocker, was evaluated in senescence-accelerated mouse prone 8 (SAMP8) animals (an AD-mouse model), and it was found that treatment with this antagonist improved social behavior and restored cognitive impairment in SAMP8 mice [297]. In the same paper, it was reported that several molecular pathways associated with NMDA functionality were altered, and that treatment with RL-208 increased p-NMDAR2B, which was associated with the induction of LTP, reduced protein levels of calpain-1 and caspase-3, and increased synaptic plasticity markers. In addition, extrasynaptic NMDAR (eNMDAR) was associated with synaptic damage in AD. Excessive eNMDAR activation can be seen as a result of increases in the extrasynaptic glutamate released from astrocytes in response to Aβ. Therefore, the selective pharmacological modulation of eNMDAR over synaptic NMDAR (sNMDAR) could be a potential target. In *3xTg*-AD mice, the use of NitroMemantine, an eNMDAR antagonist, demonstrated a significant improvement on the location-novelty object-recognition test and an increase in synaptic and dendritic density [298].

### 3.2. Potential Therapies Targeting VGCC

Novel therapeutic agents that target VGCC as part of a multitarget approach have emerged as potential treatments for AD. Selective calcium entry through L-type calcium channels was associated with increased ROS production and neuronal death [34,113]. Thus, many studies focused on novel therapeutic agents that target these channels. Recently, Michalska et al. developed a new family of 4,7-dihydro-2H-pyrazolo[3,4-b] pyridine, which function as selective blockers of L-type calcium channels but also have GSK-3b-inhibition capacity, as well as anti-inflammatory properties [299]. These new compounds were tested in in vitro models of neurodegeneration towards oxidative stress induced by rotenone, hyperphosphorylation induced by okadaic acid (OA), and cytosolic calcium concentration induced by high potassium. All the derivatives of 4,7-dihydro-2H-pyrazolo[3,4-b] pyridine exert neuroprotection against calcium overload and increased survival percentages. The most potent neuroprotectant was a compound that showed moderate VGCC antagonism and high antioxidant ability, which demonstrates the relationship between calcium entry through VGCC and increased levels of ROS. Furthermore, the compounds were tested in an ex vivo model of AD, in which one specific compound demonstrated neuroprotective effects against oxidative stress and cytosolic calcium concentration overload in OA-treated hippocampal slices [299].

Other multitarget-directed ligands have been tested in the context of AD, such as BIGI-3h, a compound that exhibits inhibitory activity against cholinesterase, MAO, GSK-3β, and calcium-channel-blockade activity through the inhibition of VGCC. Furthermore, BIGI-3h was tested in vitro, where it revealed antioxidant properties, brain penetration, and the capacity to prevent the aggregation of both tau protein and Aβ peptide [300]. Additionally, in vivo investigations with BIGI-3h (10 mg/kg intraperitoneally) showed a significant reduction in the cognitive impairment caused by scopolamine in mice assessed by the novel object-recognition test [300]. The use of calcium-channel antagonism as part of multitarget-directed ligands has been shown to have positive results in in vivo and in vitro studies so far, especially when including compounds against VGCC, suggesting the pivotal role of these channels in the pathophysiology of AD.

The use of L-type calcium-channel antagonists alone, and not as parts of multitarget-directed ligands, such as verapamil, has also been studied in AD models. Ponne et al. evaluated the protective effects of verapamil against scopolamine-induced in vitro neurotoxicity and in vivo cognitive impairment. The in vitro pre-treatment with verapamil 1 h before exposure to scopolamine protected the cells against cytotoxicity and attenuated oxidative stress and mitochondrial injury, according to the measurements of ∆Ψm. Furthermore, the pre-treatment with verapamil attenuated the downregulation of the genes involved in cholinergic function (*mACR1*), synaptic plasticity (*GAP43*, *SYP*), and calcium-dependent memory-related genes (*CREB1*, *CREBBP*, and *BDNF*) [301]. The treatment with verapamil attenuated the cognitive and behavioral deficits induced by scopolamine in mice, as measured by the elevated plus maze and passive avoidance test [301].

### 3.3. Potential Therapies Targeting TRP

#### 3.3.1. TRP Vanilloid 4

The TRP vanilloid 4 (TRPV4) is part of the TRP superfamily of ion channels. It is a calcium-permeable non-selective cation channel expressed in neurons, astrocytes, and microglia. The activation of TRPV4 has been linked to disturbances in calcium homeostasis, oxidative-stress-induced cell damage, and hippocampal cell death induced by Aβ [302]. Deng et al. recently evaluated a specific TRPV4 inhibitor, named HC067047, against scopolamine-induced cognitive dysfunction in mice. The results showed that exposure to scopolamine lead to the upregulation of TRPV4 expression in the hippocampus in the treated mice. In addition, the administration of HC067047 ameliorated scopolamine-induced cognitive dysfunction, as evaluated by the novel place-recognition test and the novel object-recognition test, and reduced the levels of proapoptotic proteins (Bax and caspase-3) while increasing the levels of neurogenesis markers (DCX and NeuN) [303].

#### 3.3.2. TRP Cation Channel/Vanilloid Receptor

The TRP cation channel/vanilloid receptor (TRPV1) is a ligand-gated non-selective cation channel permeable by calcium, usually recognized as a pain receptor, and expressed mostly in the PNS, but also in the CNS (e.g., the hippocampus and cortex). It is involved in the modulation of synaptic transmission and plasticity [304], and it is located in neurons, astrocytes, and microglia.

The endocannabinoid anandamide, which has been found to possess protective qualities against Aβ-induced neuronal damage, can activate the TRPV1 receptor. It was proposed that the protective properties of anandamide are mediated through the activation of the TRPV1 channel [305,306]. Consequently, Balleza-Tapia et al. investigated the role of TRPV1-receptor activation by capsaicin against Aβ-induced neuronal damage. The authors found that the TRPV1’s activation by capsaicin had strong protective and restorative properties by preventing and rescuing the impairment of hippocampal gamma oscillations, a cognition-relevant EEG pattern [307]. The activation of the TRPV1 receptor appears to prevent the Aβ-induced desynchronization of pyramidal-cell-action potentials and shifts in the balance of excitatory and inhibitory currents in CA3 pyramidal cells.

Furthermore, a recent study linked TRPV1 activation with autophagy induction, a process whose dysfunction has been identified in AD [308,309]. Subsequently, Wang et al. [310] evaluated the use of capsaicin to treat *3xTg* mice, to determine whether TRPV1-induced autophagy can have therapeutic effects in AD. On both a Y-maze spontaneous alteration test and a novel object-recognition experiment, the capsaicin treatment greatly improved the recognition of novel objects or novel areas, suggesting that the activation of TRPV1 improved learning and memory impairment in *3xTg* mice. Furthermore, immunoblotting showed that capsaicin decreased the expression of phosphorylated tau, and decrease 4G8+ amyloid-plaque accumulation in the brain [310].

On the other hand, the loss of TRPV1 function has been associated with better outcomes in AD models. In a triple-transgenic mouse model, *3xTg-AD^+/+^/TRPV1^−/−^* mice had better memory function and lower levels of Aβ, hippocampal calcium, and tau than *3xTg-AD^+/+^/TRPV1^+/+^* mice [311]. Furthermore, the authors evaluated the effects of capsazepine, a TRPV1 antagonist, on the production of Aβ and tau in *3xTg*-AD-derived primary neuronal cultures. They found that the use of capsazepine and a calcium chelator (BAPTA/AM) decreased the Aβ and tau levels [311].

#### 3.3.3. TRPC6

As previously mentioned, lower expressions of TRPC6 channels have been found in AD patients (see Section 3.2). The activation of this calcium-permeable non-selective cation channel was shown to exert synaptoprotective effects in APP knock-in mouse models of AD, mainly by stimulating the nSOCE pathway [312], thereby regulating the levels of intracellular concentrations of calcium. Building on this concept, Popugaeva et al. [313] searched for novel TRPC6 agonists to evaluate in AD models. One of the compounds, called piperazine, was able to protect mushroom spines from Aβ_42_-induced toxicity in vitro, and in *5xFAD* mice, it was able to restore LTP [313].

Furthermore, TRPC6 was also shown to be involved in the modulation of APP processing by regulating the γ-secretase cleavage of APP, thereby reducing the production of Aβ [314]. The activation of TRPC6 by hyperforin in iPSC-derived SAD neurons was shown to inhibit the secretion of Aβ_42_ or Aβ_40_ in a dose-dependent manner [315]. However, hyperforin is not very stable and shows low bioavailability. This prompted the development of other, similar semisynthetic molecules with better stability, such as hyperforin and tetrahydrohyperforin (IDN5706) [316]. Furthermore, tetrahydrohyperforin was shown to reduce the cholinergic markers associated with Aβ plaques and Aβ accumulation, as well as decreasing tau phosphorylation, in AD-mouse models [317,318]. Furthermore, tetrahydrohyperforin decreases the astrogliosis in APP/PS1 transgenic mice, preventing the inflammatory processes associated with Aβ burden [319]. Although it was not directly tested in an AD model, HYP9 was shown to inhibit TRPC6 downregulation and reduce astrocytic apoptosis, cytotoxicity, and inflammatory responses in mice with ischemic/reperfusion damage [320,321].

#### 3.3.4. TRP Channel Mucolipin 1

TRP channel mucolipin 1 (TRPML1) is an endosomal–lysosomal calcium channel linked with the pathogenesis of AD because of its role in regulating autophagy. It was found to be downregulated in *APP/PS1* transgenic mice, while the overexpression of this channel was associated with rescuing memory and recognition impairment and with attenuated neuronal apoptosis in *APP/PS1* transgenic mice [321]. The activation of these channels in postmortem late-onset AD (LOAD) hippocampal neurons using the agonist ML-SA1 was shown to restore endolysosomal calcium pools to normal levels, decrease endolysosomal swelling, and increase the levels of non-pathogenic fragments of APP [322]. Despite these promising results, few studies have examined TRPML1 regulation in AD models.

### 3.4. Potential Therapies Targeting P2Y1

As mentioned in Section 3.2, the astrocytic hyperactivity seen around Aβ plaques has been attributed in part to the activation of P2Y1 receptors, which are metabotropic purinergic receptors on astrocytes that cause intracellular calcium elevation [189]. The long-term inhibition of the P2Y1 receptor in AD-mouse models has shown a reduction in neuronal–astroglial network hyperactivity [190]. For instance, Reichenbach et al. used the P2Y1-receptor antagonist MRS2179 to treat *APP/PS1* mice for 6 weeks and examined in vivo cortical calcium activity using two-photon microscopy in astrocytes and neurons. The inhibition of P2Y1 normalized the cortical astrocytic network activity, reversed synaptic deficits, and restored synaptic integrity in the hippocampus. Furthermore, the same study evaluated the use of BTPU, an allosteric inhibitor of P2Y1, which also showed reduced astroglial hyperactivity [190]. Despite these promising results, to the best of our knowledge, this is the only study to have evaluated P2Y1 inhibition in AD models.

### 3.5. Potential Therapies Targeting MCU

The MCU is a crucial mitochondrial calcium channel that regulates cellular calcium homeostasis, thereby supporting neuronal function and survival. The malfunction of MCU can induce mitochondrial dysfunction, a key factor in the pathophysiology of AD [323,324,325]. The inhibition of calcium uptake by the MCU can regulate mitochondrial calcium homeostasis and ameliorate mitochondrial dysfunction. Following this line of thought, Liu et al. evaluated the use of ginkgolide K (GK), a compound extracted from *Ginkgo biloba* leaves, which has been shown to have multiple pharmacological properties, such as antioxidant, neuroprotectant, anti-inflammatory effects and, in some studies, to improve mitochondrial dysfunction [326] in the treatment of *APP/PS1* mice. The treatment with the GK showed the downregulation of MCU in the brains of *APP/PS1* mice, and it also alleviated cognitive impairment, as evaluated by water-maze tests [327]. In vitro (SH-SY5Y cells), Aβ was shown to increase the levels of MCU, which was significantly inhibited when exposed to the treatment with GK, causing a reduction in the levels of calcium in the mitochondria [326].

### 3.6. Potential Therapies Targeting PMCA

The PMCA is a high-affinity calcium pump that plays a very important role in the regulation of cytosolic calcium concentrations by pushing calcium out of cells in exchange for ATP hydrolysis. The inhibition of PMCA by Aβ and tau is seen in AD models and is correlated with neurodegeneration [328]. Therefore, the activation of PMCA has been considered as a new therapeutic target in AD. Berrocal et al. [329], identified sorcin, a calcium-binding protein, as a potential activator of PMCA, with a potential role in preventing the inhibition of PMCA by Aβ and tau. The same authors found that sorcin prevented the induction of toxicity in SH-SY5Y cells by Aβ and tau by preventing the inhibition PMCA. Furthermore, other studies have shown that the protective effects of some compounds in AD models are attributable to the activation PMCA. This is the case with methylene blue, a phenothiazine that has shown neuroprotective qualities in AD models. Nevertheless, until recently, the mechanism whereby methylene blue exerts these effects was not known. In another study by the same group, it was demonstrated that methylene blue is able to stimulate PMCA in the brain tissues of human patients diagnosed with AD and, furthermore, that it can prevent the Aβ-induced inhibition of PMCA [330].

### 3.7. Potential Therapies Targeting NCX3

The NCX3 is the third type of the NCX, an essential plasma-membrane transporter that regulates calcium homeostasis, and it is commonly found in neurons [331]. Reduced NCX3 function was associated with Aβ_1–42_-induced calcium dysregulation. while its increased levels were shown to protect cells against Aβ_1–42_-induced cell death [331]. Therefore, the activation of this transporter has been proposed as a novel therapeutic strategy. Afewerky et al. [331,332], evaluated the use of *Withania somnifera,* an indigenous plant that has been used for therapeutic purposes due to its neuroprotective qualities, such as its ability to decrease oxidative stress, its regulation of apoptosis, and its modulation of mitochondrial function [333], in *5xFAD* mice [332]. The authors found that the mice treated with *Withania somnifera* showed increased expressions of *NCX3,* reduced numbers of Aβ plaque deposits, and better cognitive performances [332].

### 3.8. Potential Therapies Targeting SOCE

Some mutations that cause FAD, such as *PS1ΔE9*, demonstrate increased ER calcium-leakage activity, leading to an increase in the neuronal SOCE (nSOCE) pathway and, consequently, an increase in intracellular calcium concentrations [334]. The inhibition of nSOCE in this context is therefore a potential therapeutic target for the treatment of AD. Chernyuk et al. evaluated the use of EVP4593, a nSOCE antagonist in hippocampal neurons transfected with *PS1ΔE9*, to study changes in morphology in synaptic spines [335]. The authors found that the neurons transfected with *PS1ΔE9* had an enhanced nSOCE in the hippocampal spines, but the addition of EVP4593 blocked the increase in the amplitude of nSOCE. Furthermore, EVP4593-treated neurons showed an increase in the percentage of mushroom spines, whose loss is related to cognitive decline [335].

On the other hand, many studies have reported positive modulations of SOCE, rather than its inhibition, in AD models [277,312]. For instance, the agonists of store-operated channels, such as TRPC6 (see Section 3.3.3), and the downregulation of ORAI2 channels have also been proposed as new therapeutic targets. Scremin et al. evaluated the role of ORAI2 channels in the modulation of SOCE in the human neuroglioma-cell line H4 and in the H4-APPswe cell line [277]. The authors found an inverse relationship between SOCE and Aβ accumulation. By using cyclopiazonic acid to activate SOCE, Aβ accumulation was decreased, whereas with the use of BTP2 to inhibit SOCE Aβ accumulation was increased. Furthermore, the downregulation of ORAI2 channels also led to a significant decrease in the accumulation of Aβ, suggesting this channel as a potential target [277].

### 3.9. Potential Therapies Targeting α7nAChR

The nAchRs are ligand-gated ion channels with many subtypes. Thanks to non-polar residues and high levels of glutamic acid, the α7nAChR is highly specific to calcium, and it is involved in the regulation of cognitive functions [336]. However, in AD, Aβ is able to bind to this receptor and inhibit its normal function. Agonists of α7nAChR have therefore been proposed as therapeutic targets in AD for their potential capacity to enhance cognitive function. For instance, Takata et al. evaluated the use of DMXBA, an α7nAChR agonist, in primary cultures of rat microglia and in a transgenic mouse model of AD (*APdE9* mice) [337]. In vitro, DMXBA increased microglial Aβ phagocytosis, while the in vivo treatment with DMXBA improved the performance of *APdE9* mice in spatial-learning and memory tasks. In addition, the long-term administration of DMXBA decreased the amount of Aβ_40_ in the brains of *APdE9* mice [337].

Despite promising preclinical evidence, clinical trials using α7nAChR agonists have not shown clinical benefits. A randomized, double-blind, placebo-controlled multicenter study that evaluated the efficacy and safety of ABT-126, a potent selective α7nAChR agonist, did not demonstrate improvements on the ADs Assessment Scale’ Cognitive Subscale (ADAS-Cog) nor did it improve secondary efficacy measures, such as cognition or function [338]. Nevertheless, the positive allosteric modulation of α7nAChR might be another possible therapeutic strategy aiming at this target. The administration of positive allosteric modulators of α7nAChR (CCMI and PNU-120596), in combination with currently approved anti-AD drugs, to scopolamine-induced memory deficits in rats was evaluated, and it was found that the combined administration of positive allosteric modulators and acetylcholinesterase inhibitors (donepezil) or NMDAR antagonists (memantine) improved performance on the novel object-recognition test (NORT), reversing the scopolamine-induced deficit [339].

### 3.10. Potential Therapies Targeting RyRs

The RyRs are calcium-releasing channels located on the ER-plasma membrane that are involved in the pathophysiology of AD, mainly due to overactivation, which results in calcium dysregulation. The pharmacological inhibition of these channels is therefore of interest as they are potential therapeutic targets. Recently, different compounds and repurposed drugs targeting RyR were evaluated in preclinical AD models, with promising results. Shi et al. for instance, evaluated the use of dantrolene, a RyR-receptor antagonist used in the treatment of malignant hyperthermia in *5xFAD* mice via intranasal and subcutaneous administration [340]. In the same study, chronic intranasal-dantrolene treatment ameliorated memory loss in *5xFAD* mice without causing serious adverse effects, and showed better brain penetration than subcutaneous administration. Furthermore, in iPSC derived from SAD and FAD patients, dantrolene exhibited neuroprotective effects by rescuing synaptogenesis, regulating intracellular calcium homeostasis, and increasing autophagy [341]. In addition to dantrolene, other compounds that inhibit RyR activity have been evaluated in AD models. Liu et al. used R-carvedilol to shorten RyR2’s opening time in *3xTG-AD* mice, and found that the R-carvedilol pretreatment rescued memory impairment and LTP deficit, as well as increasing the density of neurons in the subiculum area compared to the vehicle-treated group [342].

### 3.11. Potential Therapies Targeting IP3R

The calcium channel IP3R is expressed in the membrane of the ER, which is involved in the regulation of intracellular calcium homeostasis (see Section 3.4 for a detailed description). This receptor has been linked with the pathophysiology of AD due to the intracellular calcium overload observed in many AD models. In FAD, mutant *PS1* and *PS2* have been shown to interact with IP3R, causing stimulatory effects on its gating activity, resulting in exaggerated cellular calcium signaling [343]. Therefore, the inhibition of this channel could be a potential therapeutic target. The use of Xestospongin C, a reversible IP3R antagonist, was evaluated with *APP/PS1* AD mice, showing an improvement in cognitive behavior, as evaluated by the Y maze and the Morris water maze, a reduction in the number of Aβ plaques, and a reduction in the levels of ER-stress proteins [344]. To the best of our knowledge, this is the only study using AD models targeting IP3R.

### 3.12. Potential Therapies Targeting VDAC1

The VDAC1 is found in the mitochondrial outer membrane, and it is considered to be the mitochondrial gatekeeper because of its many functions, including metabolite exchange, apoptosis regulation, and the modulation of mitochondrial calcium uptake [345]. The Aβ can directly interact with VDAC1, affecting its normal function and increasing the opening of the mitochondrial-permeability-transition pore, resulting in the release of proapoptotic factors [346]. Thus, VDAC1 has emerged as a potential therapeutic target. Few studies have evaluated the pharmacological modulation of VDAC1 in AD models. However, one study used the flavonoid, hesperidin, to evaluate its neuroprotective effects on rat pheochromocytoma cells exposed to Aβ_25–35_ [347]. The authors of that study found that hesperidin was able to inhibit Aβ_25–35_-induced apoptosis, in part, by preventing the increase in the phosphorylation of VDAC1.

### 3.13. Potential Therapies Targeting CB1

Animal and human studies have shown that in AD, alterations occur in the expression of CB1, including early increases followed by a progressive reduction in their expression and alterations in their distribution [348]. In addition, postmortem studies revealed a decreased concentration of anandamide in AD brains, which was inversely correlated with cognitive scores and Aβ accumulation [305]. Therefore, CB agonists are undergoing exploration as possible therapeutic targets in AD. For instance, WIN 55,212-2 (a synthetic cannabinoid), which acts as an agonist on both CB receptors, has been shown to increase cell viability, augment the expression of antioxidant agents, and potentiate anti-inflammatory responses in primary cultured rat astrocytes treated with Aβ_1–42_ [349]. In mice inoculated with Aβ_1–42_ in the hippocampus, treatment with the phytocannabinoid, cannabidiol (CBD), was shown to reduce *GFAP* overexpression, as well as to decrease the expression and release of iNOS and IL-1β [350]. The observed reductions in reactive gliosis and in neurodegeneration due to CBD treatment in AD models seem to be dependent on the activation of the PPARγ pathway [351], which is also involved in calcium signaling. In addition, in human SH-SY5Y neuronal cells, CBD protected the reduction in dendritic spine density, increased the levels of CB1, and prevented neurite lesions from Aβ_1–42_ [352].

### 3.14. Potential Therapies Targeting CaMKII and CaN

Targeting the downstream proteins that are directly affected by dysregulated calcium, such as calmodulin and calmodulin-binding proteins, rather than calcium itself has also been considered as a possible therapeutic approach in AD. Two calcium-dependent enzymes, CaMKII and CaN, have been considered as potential targets. The complex protein kinase, CaMKII, has been linked with synaptic plasticity and memory formation [353]. The autophosphorylation of CaMKII is a fundamental event in LTP; reductions in autophosphorylation are related to cognitive dysfunction and appear to be altered in AD, while CaN has been associated with LTD [354]. Chen et al. evaluated the effect of SLOH, a carbazole-based cyanine fluorophore, on a *3xTg-AD* mouse model [355]. The SLOH exerted protective effects (reducing neuronal apoptosis and synaptic deficits) in part by regulating intracellular calcium and the CaMKII/CREB-signaling pathway, suggesting that the targeting of CaMKII could be a potential avenue for research. In addition, other studies found that the protective effects exerted by some drugs in AD models are due to the restoration of the CaMKII/CREB pathway [356]. However, to the best of our knowledge, there are no studies directly targeting CaMKII in AD models.

Previous studies demonstrated that Aβ-induced morphological neurodegeneration (spine loss, dendritic simplification) is mediated through CaN activation via the CaN/NFAT pathway [357]. Thus, the targeting of CaN has been evaluated in AD models. For instance, Rozkalne et al. evaluated whether, by inhibiting the CaN/NFAT pathway with FK506, the neuronal morphological changes could be improved. The results showed that after 1 week of treatment, there was an increase in dendritic spine density [358]. Furthermore, a recent study that evaluated the association between NCX2 and NCX3 levels with deficits in hippocampus-dependent learning and memory found that in *NCX2*+/− mice, there was an impaired maintenance of hippocampal LTP, which was positively correlated with increased CaN activity and was rescued by the administration of FK506. In the same study, the administration of FK506 to *APP-KI* mice also significantly restored hippocampal LTP, without altering LTP, in wild-type mice [157]. However, a recent study using tacrolimus in rats treated with streptozotocin did not show any preventive effects on neuronal loss [359].

**Figure 2:**
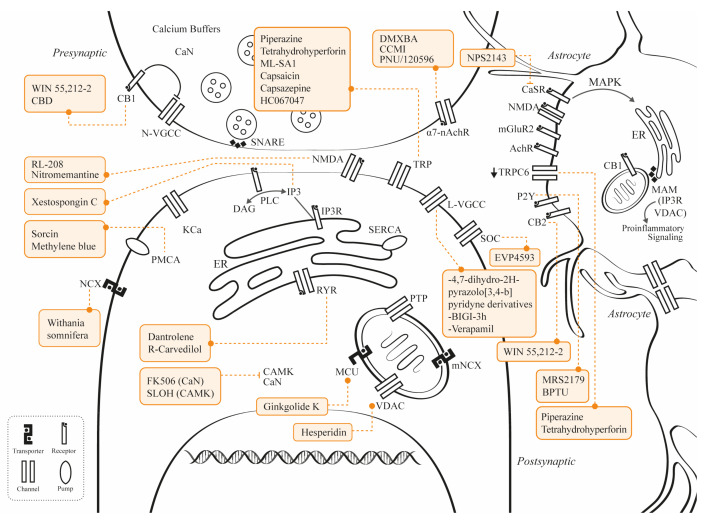
Potential therapies associated with their respective calcium targets are illustrated. Most of the molecules described here have several mechanisms of action, including anti-inflammatory, anti-amyloid, and antioxidative effects. However, they all have a main interaction with proteins involved in calcium regulation. Black arrows represent activation, and bars represent inhibition. The symbol ↓ in TRPC6, represents reduction in their expression. Abbreviations: calcium-dependent phosphatase calcineurin (CaN), calcium-sensing receptor (CaSR), cannabinoid receptor (CB), diacylglycerol (DAG), inositol trisphosphate (IP3), inositol trisphosphate receptor (IP3R), L-type VGCC (L-VGCC), metabotropic glutamate receptors (mGluR), mitochondria-associated membranes (MAMs), mitochondrial NCX (mNCLX), mitochondrial calcium uniporter (MCU), N-methyl-D-aspartate (NMDA), nicotinic acetylcholine receptors (nAChR), permeability transition pores (PTP), phospholipase C (PLC), plasma-membrane calcium-ATPases (PMCA), purinergic receptors (P2Y), ryanodine receptors (RyR), sarco/ER calcium ATPase (SER-CA), soluble N-ethylmaleimide-sensitive adhesion receptors (SNARE), sodium–calcium exchanger (NCX), store-operated Ca2+ entry (SOCE), transient receptor potential (TRP), TRP canonical channels (TRPC), voltage-gated calcium channels (VGCC), voltage dependent anion selective channel (VDAC).

A summary of potential drugs, with their respective calcium targets and mechanisms of action, is presented in Figure 2 and Table 1.

## 4. Conclusions

Preclinical data suggest the beneficial role of the regulation of calcium levels in in vitro and in vivo models of AD, by directly inhibiting either calcium channels or calcium-dependent downstream cascades. Most of the successful models so far included calcium-channel blockers as monotherapy or as part of a multitarget approach, along with antioxidants, GSK-3β inhibitors, and cholinesterase inhibitors. However, the activation of some calcium channels, such as TRPV1, TRPC6, and TRPML1, has also been shown to exert beneficial effects in acute and chronic models. Therefore, when modulating intracellular calcium levels, the molecular target and its respective cellular physiological role, the brain circuit affected, and the stage of the AD pathology should be considered. For example, a homeostatic increase in calcium levels is essential for synaptic plasticity processes in the hippocampus, but an excessive rise can induce late-onset neurodegeneration, excitotoxicity, and glial-induced neuroinflammation phenomena throughout the entire brain. The incorporation of drugs that regulate calcium dysfunction, apart from the commonly used NMDA blockers and VGCC modulators, should be considered in the future design of multitarget drugs for AD.

## Figures and Tables

**Table 1:** Summary of novel studies involving calcium-channel modulation.

Molecular Target	Therapeutic Agent	Model	Therapeutic Effect	Reference
α7nAChR	DMXBA	Primary cultures of rat microglia and transgenic mouse model (*APdE9* mice)	Increased Aβ phagocytosis	[337]
	CCMI PNU-120596	Scopolamine-induced memory deficits in rats	When used in combination with AD-approved drugs they reversed the scopolamine-induced deficit	[339]
CaN	FK506	*APP/PS* mice	Short-term treatment resulted in amelioration of dendritic spine loss	[358]
	FK506	*APP*-KI mice	Reduced LTP impairment in *APP*-KI mice	[157]
CaMKII	SLOH	*3xTG*-AD	Attenuated synaptic deficit in vivo by regulating the calcium/CaMKII/CREB-signaling pathway Reduced Aβ deposition	[355]
**CaSR**	NPS2143	Human cortical astrocytes and HCN-1A neurons	Attenuated Aβ_1–42_ secretion Reduced total CaSR protein complement Blocked excess NO production in human astrocytes Reduced the secretion of IL-6, ICAM-1	[202,203,204]
CB1	WIN 55,212-2	Primary cultured rat astrocytes treated with Aβ_1–42_	Increased cell viability and anti-inflammatory response	[349]
	CBD	Mice inoculated with human Aβ_1–42_	Reduced GFAP overexpression Reduced expression of iNOS and IL-1β	[350]
	CBD	SH-SY5Y neuronal cells	Protected the reduction in dendritic spine density, increased the expression of CB1 receptor, and prevented neurite lesions from Aβ_1–42_	[352]
IP3	Xestospongin C	*APP/PS1* mice	Improved cognitive behavior, reduced the number of Aβ plaques, and reduced the expression or ER stress proteins	[344]
L-type voltage-dependent calcium antagonist	4,7-dihydro-2H-pyrazolo[3,4-b] pyridine derivatives	SK-N-SH neuroblastoma cell line exposed to okadaic acid, rotetone, and K^+^	Improved cell viability	[299]
		Rat hippocampal slices treated with okaic acid	Reduced cellular death Reduced oxidative stress	[299]
	BIGI-3h	Scopolamine-induced cognitive dysfunction in mice evaluated with the NORT	Mice treated with BIGI-3h after scopolamine treatment showed significantly improved recognition index	[300]
	Verapamil	Human neuroblastoma cells treated with scopolamine	Attenuated the downregulation of *mACR1*, *GAP43*, *SYP*, *CREB1*, *CREBBP*, and *BDNF*	[301]
		Scopolamine-treated mice	Attenuated the cognitive and behavioral deficits induced by scopolamine	[301]
MCU	Ginkgolide K	*APP/PS1* mice	Downregulated MCU Improved cognitive ability	[327]
NCX3	*Withania somnifera*	*5xFAD* mice	Significantly improved performance on Barnes circular-maze task Increased *NCX3* expression Reduced oxidative stress	[332]
NMDA	RL-208	Senescence-accelerated mice prone 8 (SAMP8), a mouse model of late-onset AD (LOAD).	Improved social behavior and restored cognitive impairment Increased levels of p-NMDAR2B mBDNF Synaptophysin PSD95	[297]
	Nitromemantine	*3xTg-AD* mice	Improved function on the location-novelty-recognition test	[298]
PMCA	Sorcin	SH-SY5Y Cells	Preservation of PMCA activity Reduced toxicity of SH-SY5Y cells induced by Aβ	[329]
	Methylene blue	Brain tissues of human patients with AD	Activated PMCA Blocked the inhibitory effect of Aβ on PMCA activity	[330]
P2Y1	MRS2179 BPTU	*APP/PS1* mice	Decreased astrocytic hyperactivity Reversed synaptic deficits	[190]
RyR	Dantrolene	*5XFAD* mice	Ameliorated memory loss	[340]
		iPSC from SAD and FAD patients	Increased cell viability and proliferation Restored intracellular calcium homeostasis	[341]
	R-Carvedilol	*3xTg*-AD	Rescued memory impairment, LTP deficit, and neuron loss	[342]
nSOCE	EVP4593	PS1ΔE9-transfected hippocampal neurons	Rescued mushroom-spine loss	[335]
TRPC6	Piperazine	*5xFAD* mice	Restored nSOCE in hippocampal neurons Restored LTP in *5xFAD* mice	[313]
	Tetrahydrohyperforin	*AβPP/PS* transgenic mice	Decreased caspase 3 activation, tau phosphorylation, and Aβ accumulation	[318]
TRPML1	ML-SA1	Postmortem LOAD hippocampal neurons	Restored endolysosomal calcium pool to normal levels Decreased endolysosomal swelling Increased the levels of non-pathogenic fragments of APP	[322]
TRPV1	Capsaicin	Rat hippocampal slices treated with recombinant Aβ_1–42_	Prevented the reduction in hippocampal gamma oscillations	[307]
	Capsazepine	*3xTg-AD*-derived primary neuronal cultures	Decreased production of Aβ, tau, and p-tau	[311]
	Capsaicin	*3xTg* mice	Decreased the levels of phosphorylated tau Improved outcomes in NORT and on Y maze Promoted microglial autophagy	[303,310]
TRPV4	HC067047	Scopolamine-induced cognitive dysfunction in mice	Reduced scopolamine-induced cognitive dysfunction, as assessed by NPRT and NORT Reduced levels of Bax and caspase 3 Increased levels of NeuN	[303]
VDAC1	Hesperidin	Rat PC12 cells exposed to Aβ_25–35_	Reduced Aβ _25–35_-induced apoptosis	[347]

Abbreviations: mBDNF: mature brain-derived neurotrophic factor; PSD-95: postsynaptic density protein 95.

## Data Availability

Not applicable.
